# Early functional reactivity to a CEACAM5 x CD3 T cell bispecific antibody in solid tumors associates with baseline immune contexture rather than target antigen density alone

**DOI:** 10.1038/s41698-026-01643-3

**Published:** 2026-08-14

**Authors:** Sabine Wilson, Abraham Silva, Martha L. Serrano-Serrano, Vaios Karanikas, Inja Waldhauer, Anne Schoenle, Vered Bar, Seth J. Salpeter, Elisa D’Arcangelo, Matthias Lutolf, Nikolche Gjorevski, Nitya Nair

**Affiliations:** 1 Computational Sciences Center of Excellence, Roche Innovation Center Welwyn, Welwyn Garden City, UK; 2 Early Clinical Development Oncology, Roche Innovation Center Munich, Penzberg, Germany; 3 Computational Sciences Center of Excellence, Roche Innovation Center Basel, Basel, Switzerland; 4 Roche Pharmaceutical Research & Early Development, Roche Innovation Center Zurich, Zurich, Switzerland; 5 Curesponse Ltd, Rehovot, Israel; 6 Institute of Human Biology, Roche Pharma Research and Early Development, Roche Innovation Center Basel, Basel, Switzerland; 7 Roche Pharma Research & Early Development, Roche Innovation Center Basel, Basel, Switzerland

**Keywords:** Biomarkers, Cancer, Immunology, Oncology

## Abstract

Clinical translation of T cell bispecific (TCB) therapies in solid tumors is hindered by the immunosuppressive tumor microenvironment and intratumoral heterogeneity, features poorly captured by standard preclinical models. We applied an integrated translational framework, combining humanized mouse models, a patient-derived ex vivo organ culture (EVOC) platform, and Phase 1 clinical trial data (ClinTrials.gov identifier NCT03539484), to define the biologic determinants of functional reactivity to a CEACAM5 x CD3 TCB. In humanized mice bearing CEA-positive tumors, the TCB inhibited tumor growth and increased intratumoral T cell infiltration. EVOC, which preserves native 3D tumor architecture and endogenous tumor immune contexture, revealed rapid TCB-induced secretion of IFNγ, IL-2, TNFα, and IL-10 across colorectal, pancreatic, and non-small cell lung cancer patient tumors, with substantial inter-patient heterogeneity. Higher baseline CD3+ T cell infiltration trended with a greater magnitude of cytokine release in patient tumors treated with the TCB ex vivo versus tumors with lower CD3+ T cell infiltration. Baseline tumor CEA expression did not associate with functional response in EVOC. In the Phase 1 trial, on-treatment peripheral cytokine induction was greater in patients with immune-inflamed or -excluded tumors as compared to those with immune-desert tumors. EVOC recapitulated the high inter-patient functional heterogeneity observed clinically, with a coefficient of variation for IFNγ of 135% in EVOC versus 190% in patients, compared with 52% in humanized mice. These findings suggest that early pharmacodynamic responses to CEACAM5 x CD3 TCB associate with a pre-existing tumor immune state rather than target antigen expression alone and support integration of patient-derived ex vivo models for modeling clinically-relevant pharmacodynamics.

## Introduction

Immune checkpoint inhibitors have transformed outcomes for several cancers, yet most patients with solid tumors do not achieve durable benefit due to primary or acquired resistance. Multiple mechanisms, including defective antigen presentation, stromal barriers that restrict lymphocyte trafficking, and T cell exhaustion, limit the capacity of endogenous immunity to control tumor growth^1,2^. These limitations highlight the need for immunotherapies capable of bypassing dysfunctional priming and re-engaging T cells within immunosuppressive tumor microenvironments (TME)^3^.

T cell bispecific antibodies (TCBs) address these challenges by simultaneously engaging a tumor-associated antigen (TAA) and the CD3 epsilon complex on T cells, thereby triggering major histocompatibility complex–independent polyclonal T cell cytotoxicity, even in tumors with impaired antigen presentation or low neoantigen burden^4^. Contemporary 2:1 TCB formats, combining bivalently bound TAA with monovalent CD3 engagement, further enhance tumor selectivity while minimizing on-target off-tumor toxicity^5^.

CEACAM5, also known as carcinoembryonic antigen (CEA), is a cell-surface glycoprotein^6,7^, overexpressed in several solid tumors, including in colorectal cancer (CRC), pancreatic ductal adenocarcinoma (PDAC) and in a subset of non-small cell lung cancers (NSCLC), with restricted expression in normal adult tissues, making it an attractive target for antibody-based therapies. 2:1 CEACAM5 x CD3 TCBs demonstrate potent antigen-dependent T cell activation, cytokine secretion, and tumor cell killing in vitro and in vivo^8^. Furthermore, combining the CEA-targeted TCBs with FAP-4-1BBL, a fibroblast activation protein (FAP) targeted 4-1BB ligand agonist, delivers a robust, localized “Signal 2” to augment cytotoxic T cell activity, particularly within stromal compartments rich in FAP-positive cancer-associated fibroblasts^9–11^. Nevertheless, clinical development of 2:1 CEACAM5 x CD3 TCB has been constrained by on-target off-tumor toxicities, heterogeneous antigen expression and difficulty in achieving sufficient intratumoral T cell engagement while limiting toxicity^12^. Since TCB activity is dependent on antigen availability and T cell accessibility, preclinical models that fail to recapitulate these features in a clinically relevant context may inadequately predict drug activity.

Humanized mouse models enable evaluation of TCB-induced cytokine release and T cell infiltration but lack complete immune reconstitution, possess murine stromal architecture and offer limited representation of inter-patient heterogeneity^13^. In contrast, patient-derived tumor explants partially overcome these limitations by preserving native 3D architecture, antigen expression patterns, stromal composition, and, importantly, endogenous tumor-infiltrating lymphocytes, positioning them as an important translational model for immune therapies^14^. In fact, early ex vivo T cell reactivation in patient-derived tumor fragments has been shown to correlate with clinical responses to immune checkpoint blockade in solid tumors^15,16^.

We employed an integrated translational framework—combining humanized mouse models, a patient-derived ex vivo organ culture (EVOC) model, and Phase 1 clinical trial data (ClinTrials.gov identifier NCT03539484)—to define the biologic determinants of early pharmacodynamic (PD) responses to a 2:1 CEACAM5 x CD3 TCB. Our findings demonstrate that baseline immune contexture, rather than target antigen density alone, is a key determinant of functional TCB activity in solid tumors and suggest that EVOC recapitulates clinically meaningful inter-patient functional heterogeneity.

## Results

### Integrated translational framework for evaluating CEACAM5 x CD3 TCB pharmacodynamics

**Fig. 1:**
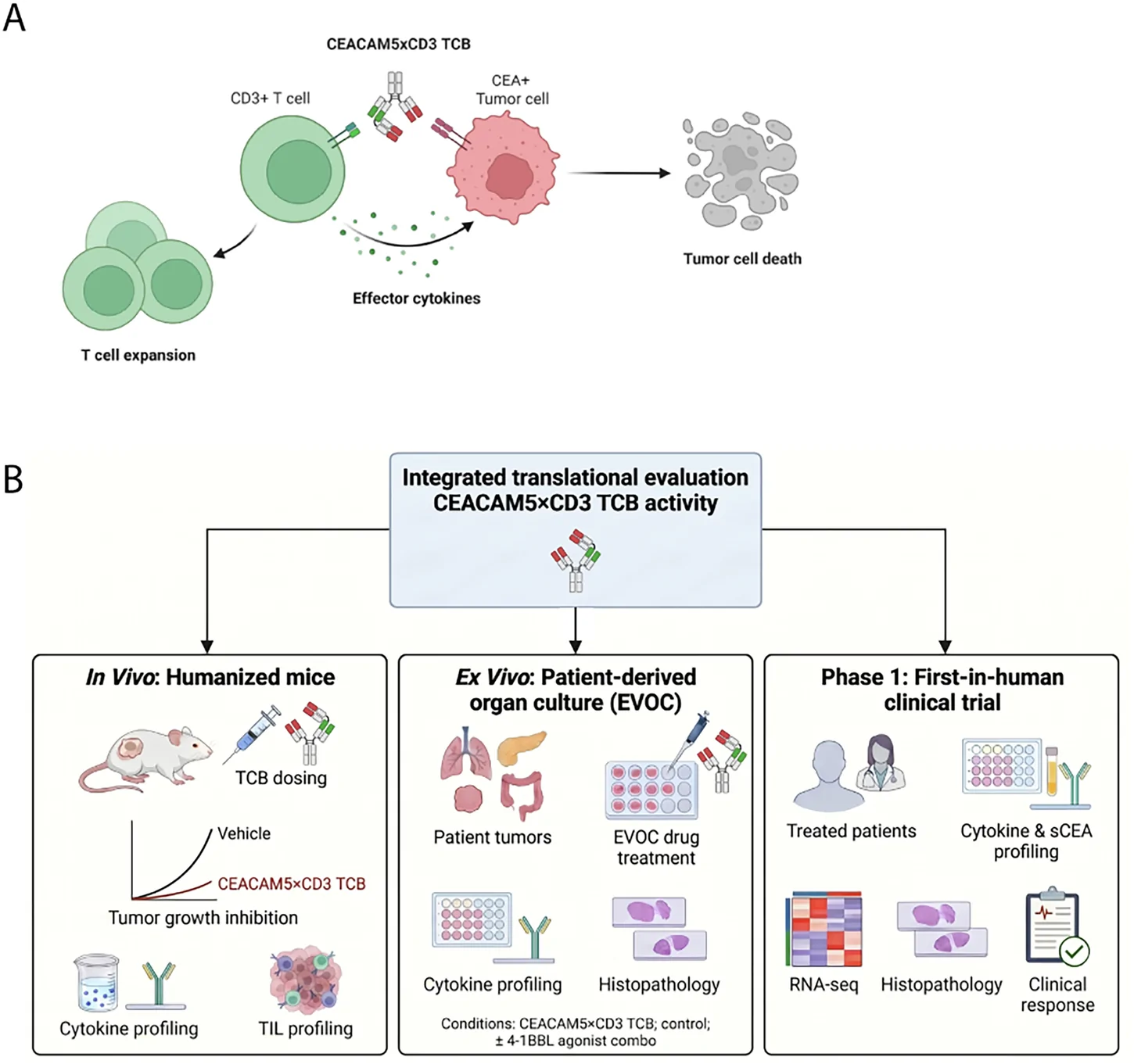
Integrated translational framework for evaluating CEACAM5 x CD3 TCB pharmacodynamics. **A** Schematic of CEACAM5 x CD3 T cell bispecific antibody mechanism of action, illustrating tumor cell engagement, CD3-mediated T-cell activation, cytokine release and tumor cell killing. **B** Overview of the integrated experimental framework combining in vivo humanized mouse studies, ex vivo patient-derived tumor explants and translational biomarker analyses from a Phase 1 clinical trial of CEACAM5 x CD3 TCB (ClinTrials.gov identifier NCT03539484).

An integrated experimental framework was established to evaluate the biological determinants of CEACAM5 x CD3 TCB-induced PD activity. The mechanism of action of CEACAM5 x CD3 TCB, including tumor cell engagement, T cell activation, cytokine release and tumor cell killing, is schematized in Fig. 1A. By coupling humanized murine models with a patient-derived EVOC platform and Phase 1 clinical trial samples, we aim to resolve how the native human TME influences early drug-induced pharmacodynamics (Fig. 1B).

### CEACAM5 x CD3 TCB induces tumor control and intratumoral immune activation in humanized mice

**Fig. 2:**
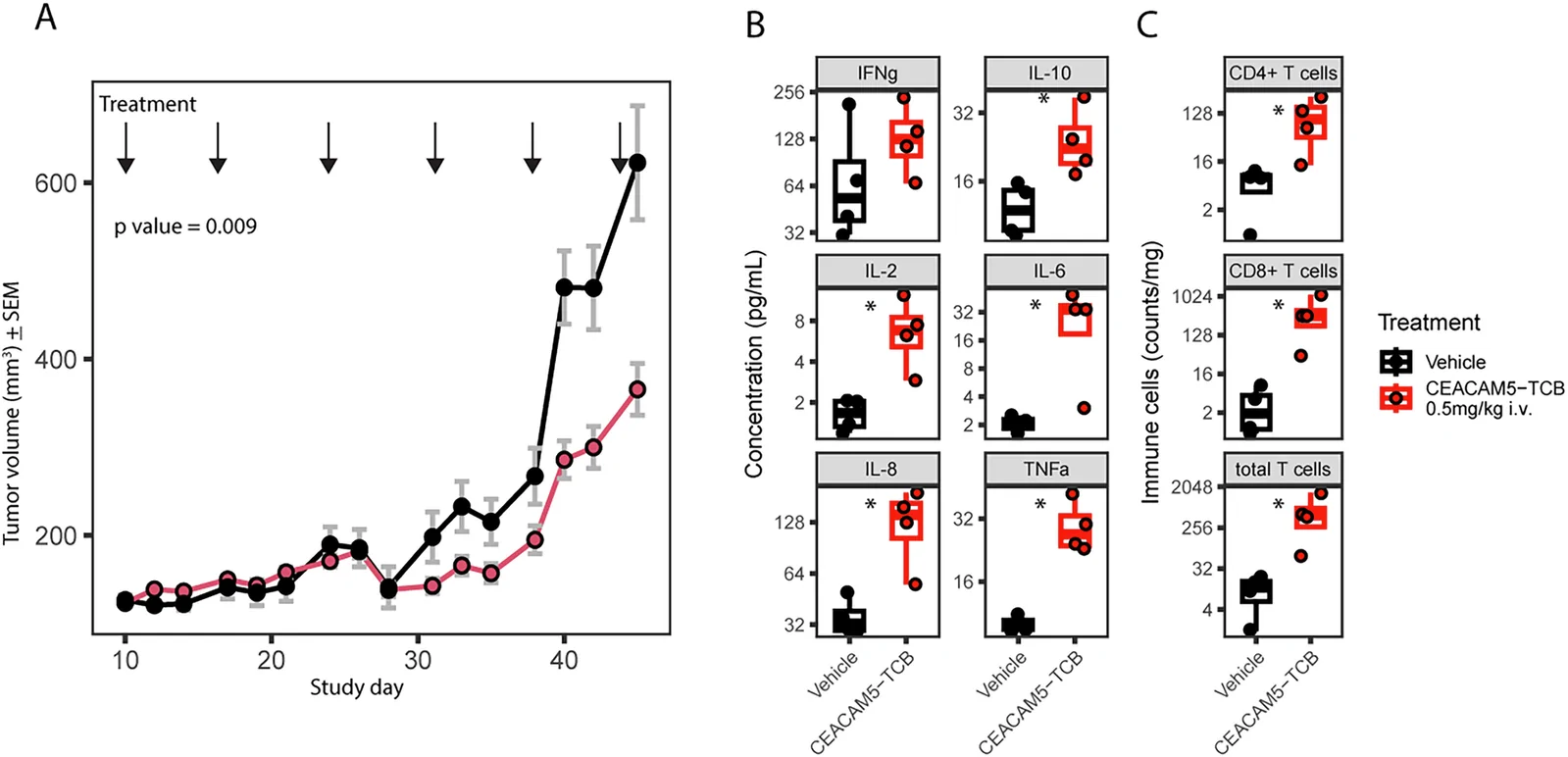
CEACAM5 x CD3 TCB induces tumor growth inhibition and intratumoral immune activation in humanized mice. **A** Tumor growth curves of CEA-positive MKN-45 xenografts in humanized mice treated with CEACAM5 x CD3 TCB (0.5 mg/kg, intravenous, weekly) or vehicle control comprised of histidine buffer (*n* = 10 mice per group). **B** Intratumoral cytokine concentrations measured 24 h by Bio-Plex Luminex assays, after the second dose, showing significant increases in IL-2, TNFα, IL-6, IL-8 and IL-10, with a numerical increase in IFNγ (*n* = 4 mice per group). Concentrations are shown on log2-scaled y-axis. **C** Quantification of intratumoral CD3+, CD4+, and CD8+ T cell infiltration 24 h after the second dose (post-treatment) compared to vehicle control (histidine buffer) by flow cytometry (*n* = 4 mice per group). Absolute T cell counts are expressed as counts per mg of tumor tissue. Briefly, harvested tumors (or representative fragments thereof) were weighed prior to mechanical and enzymatic dissociation. Total viable cell counts obtained via flow cytometry were then normalized to the recorded tissue weight to allow for cross-sample comparison of infiltration density. Data are shown as individual values with summary statistics; The y-axis represents cytokine concentrations transformed to a log2 scale to allow for visualization of broad dynamic ranges while maintaining the relative magnitude of induction. Statistical comparisons were performed using the Wilcoxon signed-rank test. *P < 0.05.

The activity of CEACAM5 x CD3 TCB was first evaluated in humanized NOD scid gamma (NSG) mice bearing established CEA-positive MKN-45 gastric adenocarcinoma tumors. Weekly intravenous (i.v.) dosing at 0.5 mg/kg resulted in significantly lower tumor volume compared to the vehicle (histidine buffer)-treated animals (*p* = 0.0093) with a tumor growth inhibition of 57% (Fig. 2A).

Intratumoral PD was assessed 24 h after the second dose to maximize detection of T cell infiltration. CEACAM5 x CD3 TCB treatment significantly increased intratumoral levels of multiple cytokines in treated mice, including IL-2, TNFα, IL-6, IL-8 and IL-10, with IFNγ showing a numerical increase as compared to the vehicle control (Fig. 2B). The distribution of cytokine data, assessed by the coefficient of variation (CV), was 52% for IFNγ with a range of 30–64% across cytokines (Fig. 2B and Supplementary Table S1). These intratumoral cytokine changes were accompanied by increased infiltration of intratumoral CD3⁺, CD4⁺ and CD8⁺ T cells relative to vehicle control-treated animals (Fig. 2C), confirming on-target immune activation in vivo.

### Patient-derived tumor explants preserve native TME architecture, CEA expression and immune cell infiltration

**Fig. 3:**
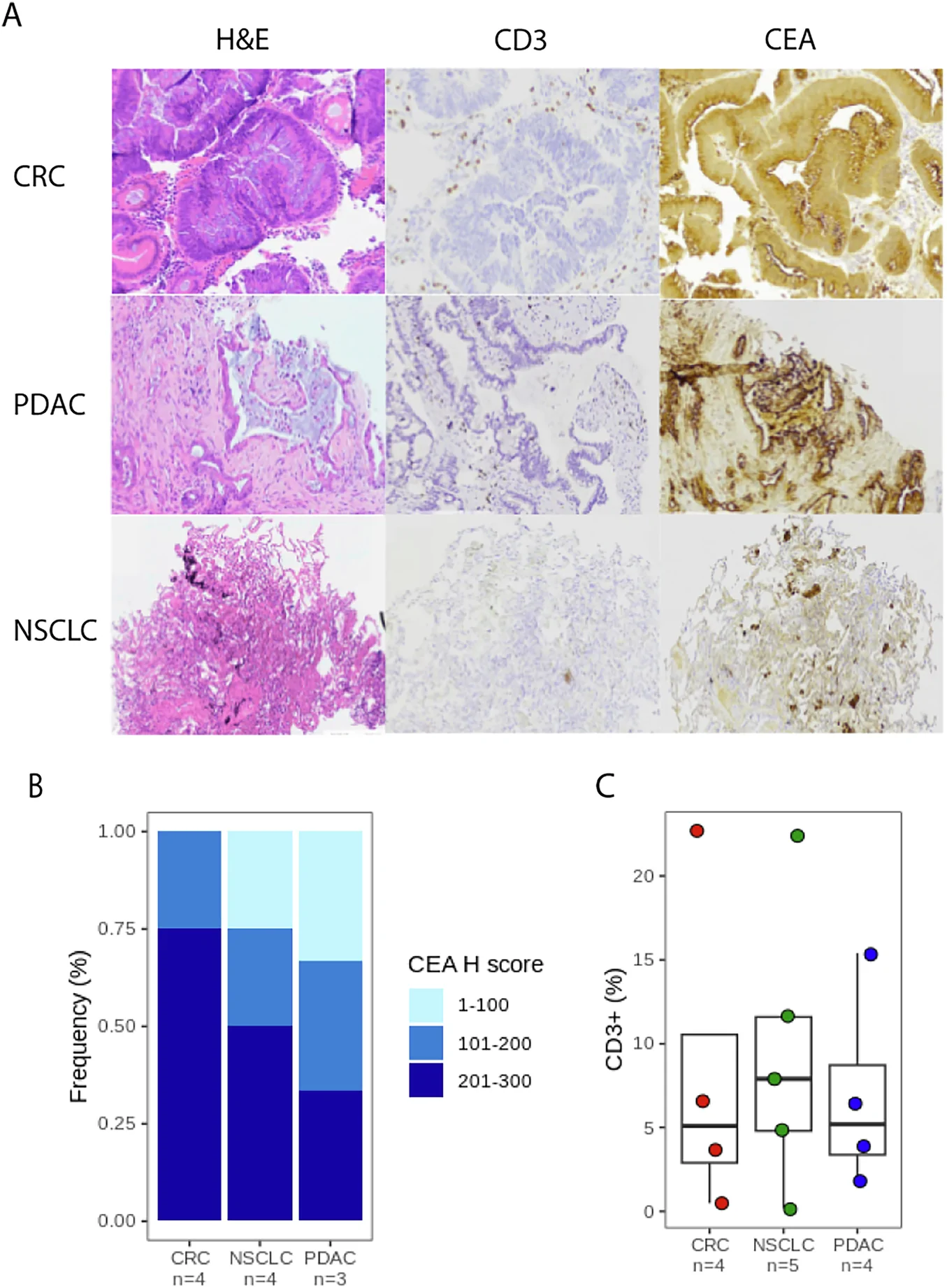
Baseline characterization of patient-derived tumor explants. **A** Representative serial sections across three distinct tumor indications demonstrate highly consistent tissue architecture and precise spatial correlation across hematoxylin and eosin (H&E), CD3 and CEA immunohistochemistry stains. Micrographs illustrate the structural alignment of tumor nests, microenvironmental boundaries and biomarker profiles within moderately differentiated colorectal carcinoma (CRC), non-small cell lung cancer (NSCLC) and pancreatic ductal adenocarcinoma (PDAC) patient-derived tumor explants prior to treatment, confirming morphological fidelity between standard H&E, CD3 + T cell infiltration patterns, and epithelial CEA expression for each independent case. **B** Baseline CEA expression across tumor indications, quantified by semiquantitative H-score (*n* = 11). **C** Percentage of intratumoral CD3+ was calculated as the number of CD3 positive cells divided by the total number of detected cells within the analyzed region of interest quantified by QuPath software, across tumor indications at baseline (*n* = 13).

To assess CEACAM5 x CD3 TCB activity within a preserved human TME morphology, tumor specimens from 14 patients with CRC, PDAC, and NSCLC were cultured ex vivo using an EVOC model previously developed by Gavert et al.^17^. Baseline clinicopathologic features of patient tumors, including tumor indication, histologic subtype, intratumoral CD3⁺ T cell density and CEA expression assessed by H-score, are summarized in Table 1. Histologic assessment confirmed that the patient's tumor native intratumoral stromal composition, architecture, and endogenous immune contexture were preserved in EVOC at baseline (Fig. 3A). Consistent with prior reports for CRC, PDAC and NSCLC^18,19^, all tumors were CEA positive, with CRC demonstrating the strongest and most homogeneous expression, followed by PDAC and NSCLC. NSCLC tumors tended to have higher CD3 T cell infiltration as compared to PDAC or CRC, both of which exhibited lower CD3 T cell infiltration, although not statistically significant^20^. Both CEA expression and baseline CD3⁺ T cell infiltration exhibited substantial inter-patient variability (Fig. 3B, C and Table 1; CEA H-score range 10 to 285; CD3% range 0.2–22.7). Short-term culture in EVOC up to 72 h maintained tissue viability, proliferative capacity and stable CEA expression (Supplementary Fig. S1).

**Table 1:** Overview of patient tumor samples used in EVOC

No.	Indication	Origin	Histologic subtype	CD3%	CEA *H*-score
1	CRC	Primary	Adenocarcinoma	0.5	10
2	CRC	Primary	Adenocarcinoma	22.7	215
3	CRC	Primary	Adenocarcinoma	3.7	190
4	CRC	Metastatic	Adenocarcinoma	6.5	245
5B273	CRC	NA	Adenocarcinoma	NA	224
6	NSCLC	Primary	Adenocarcinoma	0.2	NA
7	NSCLC	Primary	Adenocarcinoma	22.4	120
8	NSCLC	Primary	Adenocarcinoma	4.8	80
9	NSCLC	Primary	Squamous	11.6	285
10	NSCLC	Primary	Adenocarcinoma	7.9	255
11	PDAC	Primary	Ductal Adenocarcinoma	1.8	80
12	PDAC	Primary	Ductal Adenocarcinoma	3.9	190
13	PDAC	Primary	Ductal Adenocarcinoma	15.3	NA
14	PDAC	Primary	Ductal Adenocarcinoma	6.5	235

*CRC* colorectal cancer, *PDAC* pancreatic ductal adenocarcinoma, *NSCLC* non-small cell lung cancer, *NA* not available, *CEA* carcinoembryonic antigen.

### CEACAM5 x CD3 TCB-mediated cytokine responses ex vivo tend to be higher in tumors with higher T cell infiltration and do not associate with baseline CEA expression

**Fig. 4:**
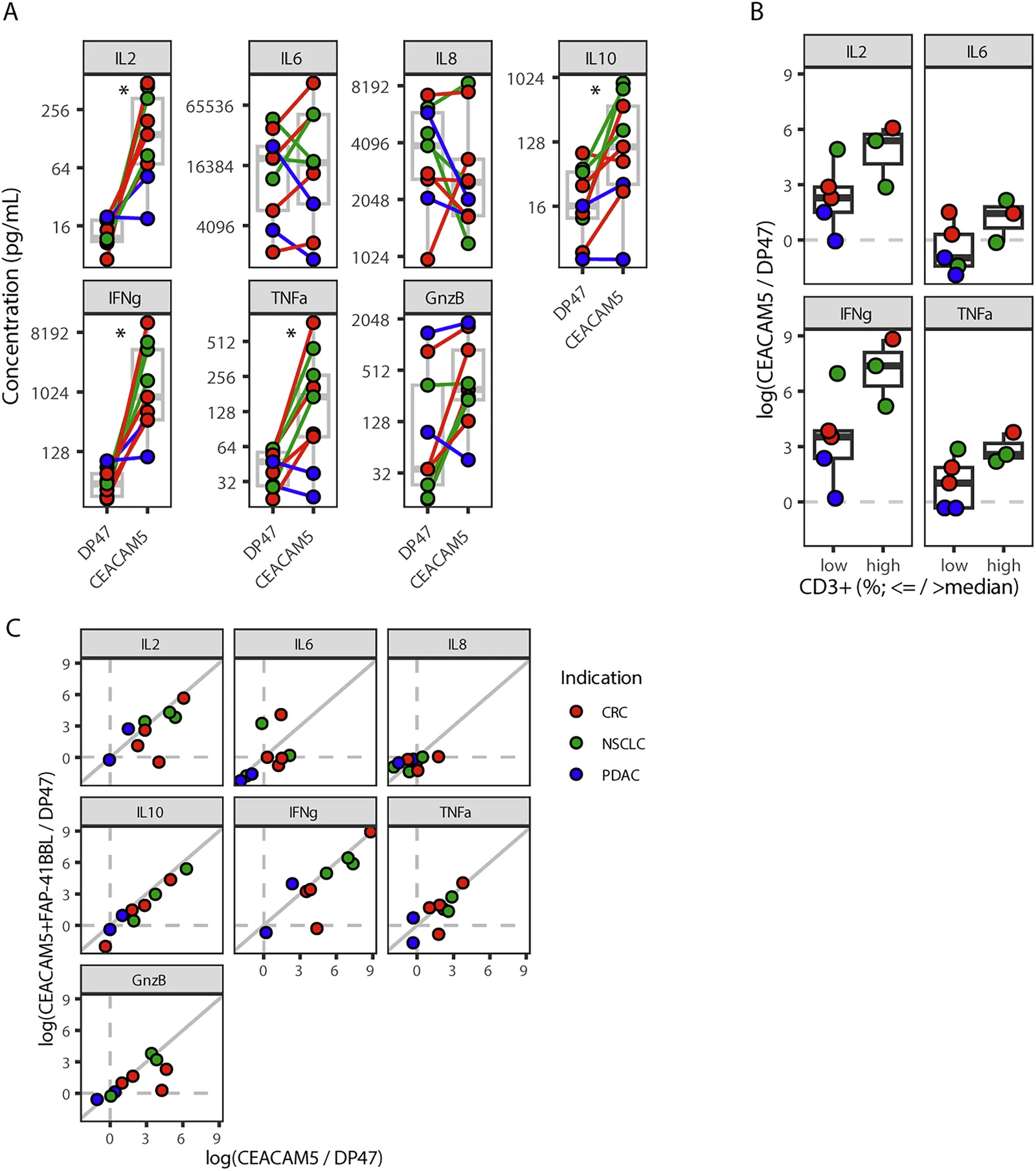
CEACAM5 x CD3 TCB elicits rapid, T cell–dependent cytokine responses in ex vivo organ culture of patient tumors. **A** Cytokine concentrations in supernatants obtained from ex vivo organ culture (EVOC) of patient tumors at 48 h following treatment with CEACAM5 x CD3 TCB compared with DP47 × CD3 TCB untargeted control, quantified by Procarta Plex Luminex assays, demonstrating significant induction of IFNγ, IL-2, IL-10 and TNFα, with a modest increase in Granzyme B (*n* = 9 tumors). Concentrations are shown on a log2-scaled y-axis. **B** Comparison of cytokine induction at 48 h, expressed as log-transformed treatment-to-control ratios (TCR), between tumors with high and low baseline intratumoral CD3+ T cell density. **C** Comparison of cytokine induction following CEACAM5 x CD3 TCB monotherapy versus combination with FAP-4-1BBL, expressed as log-transformed TCR at 48 h. The y-axis represents cytokine concentrations transformed to a log2 scale to allow for visualization of broad dynamic ranges while maintaining the relative magnitude of induction. Statistical significance was assessed using the Wilcoxon signed-rank test for paired comparisons and Spearman rank correlation for association analyses; color indicates indication; colorectal cancer (CRC), pancreatic ductal adenocarcinoma (PDAC) or non-small cell lung cancer (NSCLC).

Exposure of patient-derived tumor tissue to CEACAM5 x CD3 TCB ex vivo for 48 h induced robust cytokine responses compared with an untargeted control (DP47 × CD3 TCB). Significant increases were observed for IFNγ, IL-2, IL-10 and TNFα, while Granzyme B showed a modest, non-significant increase (Fig. 4A). A substantial level of inter-patient heterogeneity was observed in cytokine response with CVs ranging from 78% to 135% across cytokines (Fig. 4A and Supplementary Table S1). Although not statistically significant, higher baseline CD3⁺ T cell infiltration trended with a greater magnitude of cytokine release in EVOC samples as compared to samples with lower CD3+ T cell infiltration, with a high degree of inter-patient heterogeneity (Fig. 4B). In contrast, baseline CEA expression did not associate with functional responses to CEACAM5 x CD3 TCB ex vivo (Supplementary Fig. S2).

Based on preclinical data, we hypothesized that adding FAP-4-1BBL to CEACAM5 x CD3 TCB would provide a potent, costimulatory signal (“Signal 2”) that enhances T cell activation and, consequently, functional reactivity of the TCB. However, the addition of a FAP-4-1BBL bispecific antibody did not enhance cytokine induction relative to CEACAM5 x CD3 TCB monotherapy ex vivo (Fig. 4C).

### Baseline tumor characteristics and pharmacokinetics in Phase 1 patients

**Fig. 5:**
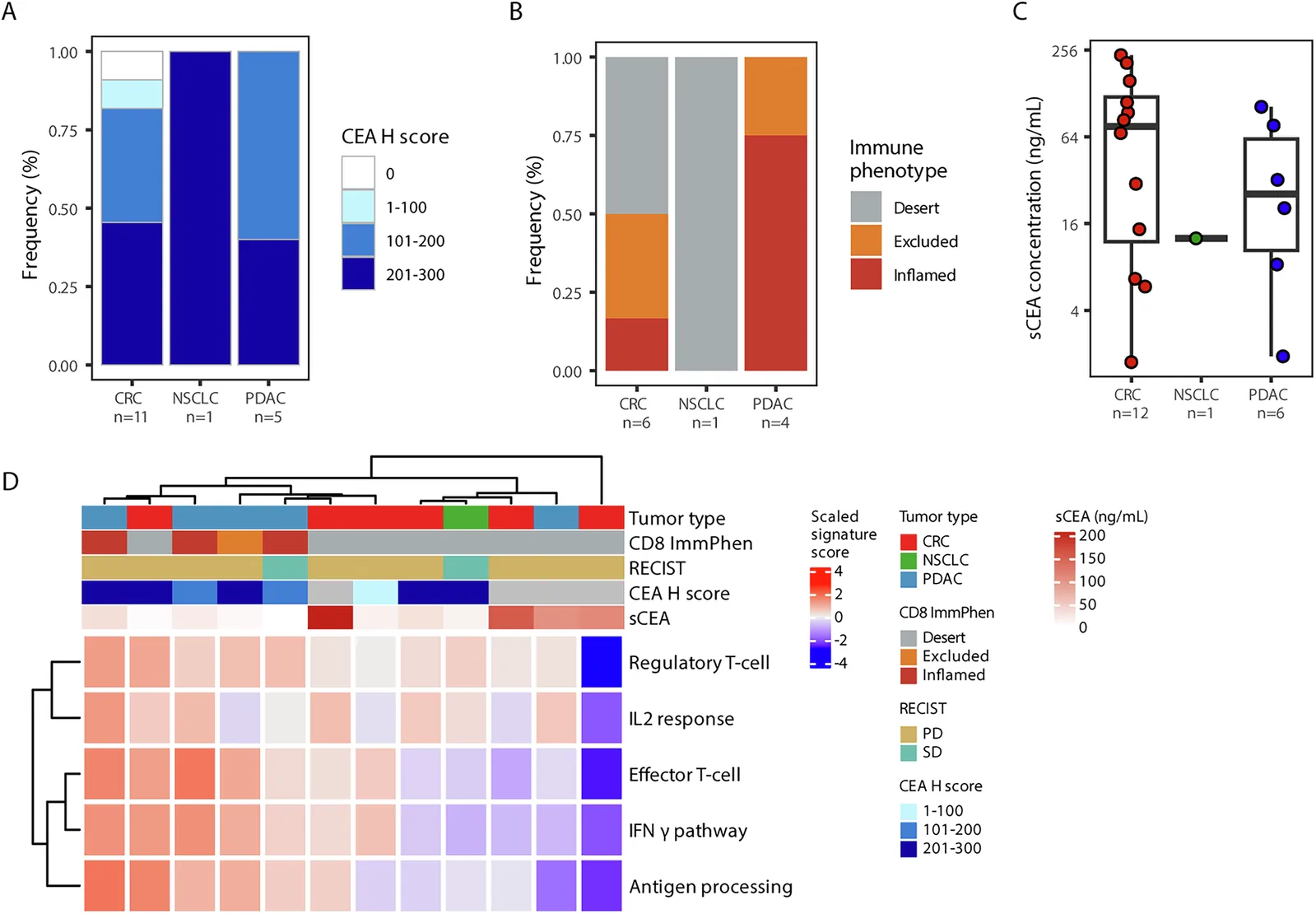
Baseline tumor and circulating biomarkers in colorectal cancer (CRC), pancreatic ductal adenocarcinoma (PDAC) and non-small cell lung cancer (NSCLC) patients enrolled in a Phase 1 study of CEACAM5 x CD3 TCB. Baseline characterization of patient tumors and circulating biomarkers. **A** Baseline tumor CEA expressed as H-scores (*n* = 17). **B** Distribution of CD8-defined immune phenotypes categorized by spatial distribution of T cells in baseline tumor biopsies (inflamed: high infiltration within tumor epithelial nests, excluded: T cells restricted to the intratumoral stroma with minimal epithelial infiltration, desert: minimal T cell presence in both epithelial and stromal compartments) (*n* = 11). **C** Baseline soluble CEA (sCEA concentrations measured in plasma (*n* = 19). **D** Integrated heatmap to visualize baseline features per patient, including tumor microenvironment profiles showing higher interferon-γ–responsive and antigen presentation pathway activity in patients with immune-inflamed and excluded tumors as compared to desert tumors, alongside baseline sCEA, tumor CEA, peripheral CD3+ T cell levels and objective response rate (ORR).

We benchmarked the in vivo and ex vivo findings with translational biomarker data from 18 CRC, PDAC, and NSCLC patients enrolled in a Phase 1 dose-escalation study of CEACAM5 x CD3 TCB (ClinicalTrials.gov identifier NCT03539484). Baseline clinical, tumor and circulating biomarker characteristics, including dose level, tumor immune phenotype, intratumoral CD8⁺ T-cell density, tumor CEA expression and baseline soluble CEA (sCEA) concentrations, are detailed in Table 2. Prior to treatment, patient tumors exhibited high CEA expression with H-scores >100 across nearly all patients evaluated (Fig. 5A), and diverse tumor immune phenotypes (Fig. 5B). Classification of tumors into immune-desert, excluded or inflamed phenotypes was based on intratumoral CD8⁺ T cell density (Supplementary Fig. S3). Similar to the patient tumors evaluated in EVOC, 50% of baseline tumors from patients in the Phase 1 study had high CEA expression (H-score >200 for 8 of 17 patient tumors from the Phase 1 study, 6 of 11 patient tumors evaluated in EVOC). Variable levels of baseline sCEA were observed across the patient cohort (Fig. 5C). Bulk RNA sequencing of patient tumor biopsies at baseline demonstrated that immune-inflamed and excluded tumors were characterized by higher baseline expression of IFNγ–responsive, effector T cell signatures and antigen presentation pathways as compared to immune-desert tumors (Fig. 5D).

**Table 2:** Overview of patient cohort from the Phase 1 study of CEACAM5 x CD3 TCB

No.	Indication	Histologic subtype	Dose (μg)	Immune phenotype	CD8 density (mm^2^)	CEA H-score	sCEA (ng/mL)	sCEA category
1	CRC	Adenocarcinoma	400	NA	NA	300	94.3	Medium
2	CRC	Adenocarcinoma	800	Desert	24.5	280	111.1	High
3	CRC	Adenocarcinoma	800	NA	NA	298	236	High
4	CRC	Adenocarcinoma	1200	NA	NA	90	14.6	Low
5	CRC	Adenocarcinoma	400	Excluded	231.1	203	30.2	Low
6	CRC	Adenocarcinoma	1200	NA	NA	245	83.6	Medium
7	CRC	Adenocarcinoma	800	Desert	9.4	295	208	High
8	CRC	Adenocarcinoma	800	NA	NA	243	156.1	High
9	CRC	Adenocarcinoma	800	Desert	57.4	290	6.6	Low
10	CRC	Adenocarcinoma	1200	Excluded	574.4	NA	1.8	Low
11	CRC	Adenocarcinoma	800	NA		299	5.9	Low
12	CRC	Adenocarcinoma	1200	Inflamed	2142.5	NA	67.9	Medium
13	PDAC	Ductal adenocarcinoma	800	Inflamed	1630.1	159	1.9	Low
14	PDAC	Ductal adenocarcinoma	800	Inflamed	356.4	240	76.7	Medium
15	PDAC	Ductal adenocarcinoma	1200	Excluded	403.8	248	8.3	Low
16	PDAC	Ductal adenocarcinoma	800	NA	NA	230	32.2	Low
17	PDAC	Ductal adenocarcinoma	1200	Inflamed	3002.3	NA	20.5	Low
18	NSCLC	Adenocarcinoma	800	Desert	39.8	300	12.6	Low

*CRC* colorectal cancer, *PDAC* pancreatic ductal adenocarcinoma, *NSCLC* non-small cell lung cancer, *NA* not available, *CEA* carcinoembryonic antigen, *sCEA* soluble CEA.

Pharmacokinetic (PK) analyses demonstrated that patients with medium (71–100 ng/mL) or high (>100 ng/mL) baseline sCEA—that could sequester drug, acting as a decoy or “sink”—had reduced active drug exposure at lower dose levels, while adequate exposure was maintained in patients with low sCEA (<70 ng/mL) or at higher doses (Supplementary Fig. S4).

### Systemic cytokine induction reflects baseline tumor immune contexture and drug exposure in Phase 1 patients

**Fig. 6:**
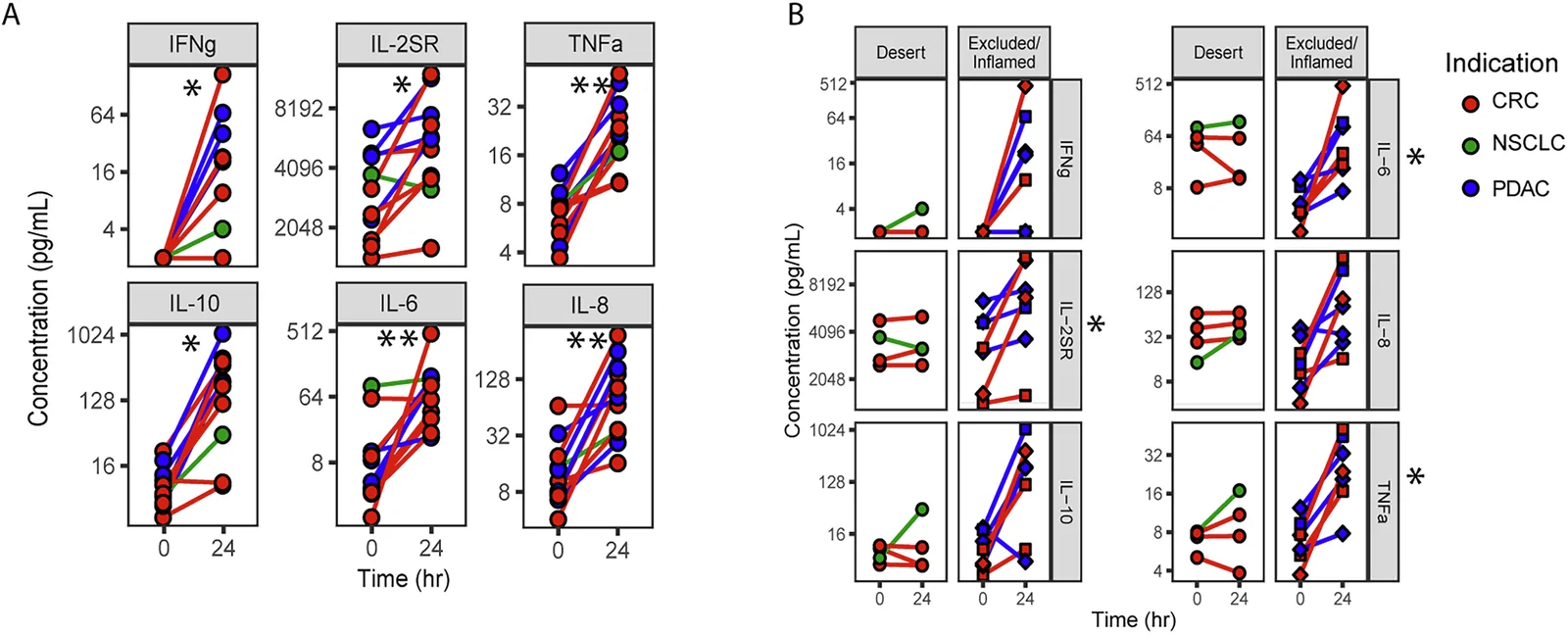
Systemic pharmacodynamic responses reflect baseline immune contexture and drug exposure in colorectal cancer (CRC), pancreatic ductal adenocarcinoma (PDAC), and non-small cell lung cancer (NSCLC) patients (*n* = 19) enrolled in a Phase 1 dose-escalation study of CEACAM5 x CD3 TCB. **A** Peripheral cytokine concentrations at baseline and 24 h after CEACAM5 x CD3 TCB administration in patients with low baseline soluble CEA (sCEA) (*n* = 9), showing significant induction of IFNγ, TNFα, soluble IL-2 receptor (IL2SR), IL-10, IL-6 and IL-8. **B** Peripheral cytokine induction at 24 h stratified by baseline tumor immune phenotype (*n* = 11), demonstrating significantly greater responses in immune-inflamed and excluded tumors compared with desert tumors for TNFa, IL-2 receptor and IL-6. Peripheral cytokines quantified using the ProteinSimple Ella platform. Concentrations are shown on log2-scaled y-axis.

In the small cohort of 18 patients evaluated from the Phase 1 study, peripheral IFNγ concentrations increased rapidly following CEACAM5 x CD3 TCB administration and were most pronounced in patients with low baseline sCEA following the first dose (Supplementary Fig. S5). At 24 h post-dose, significant increases were observed in peripheral levels of IFNγ, TNFα, IL-2SR, IL-10, IL-6, and IL-8, compared with pre-treatment levels (Fig. 6A and Supplementary Fig. S5). Similar to ex vivo findings, a substantial level of inter-patient heterogeneity was observed in cytokine responses at 24 h post-dose (CVs ranging from 79 to 190% across cytokines; Supplementary Table 1).

Stratification by baseline tumor immune phenotype revealed significantly higher peripheral cytokine induction (IL2SR, IL-6, and TNFɑ) in patients with immune-inflamed or excluded tumors compared to patients with immune desert tumors (Fig. 6B). A similar trend was observed for IFNγ although not statistically significant. These clinical observations, including the high inter-patient heterogeneity in cytokine response, were consistent with the trends observed in EVOC. In contrast, baseline CEA expression or peripheral CD3+ T cell abundance did not correlate with systemic cytokine induction (Supplementary Figs. S6 and S7), consistent with ex vivo observations. It should be noted that no significant clinical responses were observed in any patients on trial (Fig. 5D).

### No significant changes in intratumoral gene signatures and proteins on-treatment in Phase 1 patients

To evaluate the effects of CEACAM5 x CD3 TCB treatment in situ, paired tumor biopsies obtained prior to treatment and at Cycle 2 Day 8 (pre-dose) were evaluated. No significant changes were observed in TME immune or non-immune pathways based on transcriptional analysis or immunohistochemistry, including cytotoxic T cell signatures, IFNγ pathway activity or CEA expression (Supplementary Figs. S8, S9, S10 and Table S2).

## Discussion

The clinical translation of TCBs in solid tumors remains challenging due to the highly complex, immunosuppressive TME and significant inter-patient heterogeneity. In this study, we demonstrate that patient-derived tumor explants can model clinically-relevant early PD responses to CEACAM5 x CD3 TCB, in particular by reflecting the extent of inter-patient functional heterogeneity. In the CRC, PDAC and NSCLC tumors evaluated, CEACAM5 x CD3 TCB induced rapid proinflammatory cytokine responses ex vivo that closely mirrored the early effect size and broad variability of cytokine responses observed in patients treated with CEACAM5 x CD3 TCB in a Phase 1 clinical trial (IFNγ CV: 135% in EVOC vs. 190% in Phase 1). The low variability in cytokine responses observed in vivo in humanized mouse models (IFNγ CV: 52%) compared to patient-derived samples suggests that in vivo mouse models, while useful for proof-of-concept, significantly underestimated the inherent heterogeneity in patient functional responses. The preserved native 3D tissue architecture, heterogeneity and endogenous immune contexture of patient tumors in EVOC may enable improved estimation of early PD effect size aiding translatability in the clinic^21^.

A central finding across both ex vivo and clinical datasets is that early systemic PD responses associate with pre-existing tumor immune state rather than baseline tumor CEA expression alone. These results are consistent with the broader clinical and translational experience with TCBs as well as antibody drug conjugates^22^. While antigen expression is required to enable T cell redirection, multiple studies indicate that it is insufficient to predict the magnitude of immune activation in solid tumors, where baseline immune contexture, effector cell accessibility and additional TME features play dominant roles^23–25^. Within patient populations enriched based on target expression, as was the case in this study e.g., by indication selection, these factors appear to outweigh differences in target density, providing a plausible explanation for the limited association between CEA expression and functional response observed in both our ex vivo and clinical cohort analysis in CRC, NSCLC and PDAC patients. Under these conditions, antigen availability is unlikely to be rate-limiting for early T cell engagement, and other variables such as immune contexture and drug exposure, influenced by sCEA in the case of patients on trial, drive early functional reactivity. This interpretation is also supported by data from clinical trials of Cibisatamab, where baseline T cell infiltration and inflammatory gene signatures tended to associate with PD rather than CEA expression alone^26^. Similarly, Teijeira and colleagues^27^ highlight that effective T cell redirection in solid tumors depends on both target engagement and a permissive immune microenvironment, with antigen expression acting as an enabling but not the sole driver.

The robust induction of systemic cytokines in the absence of significant downstream intratumoral changes raises questions regarding the site of T cell engagement. While it is possible the TCB is partially sequestered by circulating human PBMCs or acts as a systemic CD3 agonist, our data suggest that peripheral cytokine responses are associated with baseline tumor T cell infiltration, rather than peripheral T cell levels. Further investigation into specific immune features, such as the trend for higher IL-6 and IL-8 in immune desert tumors compared to inflamed tumors, which may be related to the presence of immunosuppressive myeloid cells, could be evaluated in future studies^28,29^.

Beyond the baseline immune landscape, our data underscore circulating soluble antigen as a dominant PK determinant of TCB exposure and subsequent PD that could not be effectively modeled ex vivo. Baseline sCEA serves as a peripheral “sink”, binding the CEACAM5 x CD3 TCB and reducing its effective concentration before it can reach the TME, especially at lower dose levels. Patients with low sCEA demonstrated the most robust cytokine induction because they maintained higher levels of active, unbound TCB, capable of productive T cell engagement. These data suggest that sCEA levels are a critical component for systemic drug exposure, which in turn is a prerequisite for robust PD.

Interestingly, despite clear evidence of early systemic PD, paired clinical biopsies revealed no changes in downstream IFNγ gene signatures, CEA expression, or other pathway activation at later timepoints. Early cytokine responses may reflect activation of pre-existing T cells that may, in turn, require additional signals and a more sustained localized activation threshold to induce downstream TME changes. These data are in line with the lack of clinically significant anti-tumor efficacy observed in the Phase 1 trial at the dose levels evaluated. Due to early termination of the study, it was not feasible to assess the efficacy of CEACAM5 ⤬ CD3 TCB at biologically active dose levels, limiting a thorough investigation into the relationship between exposure and distal PD in the TME.

Contrary to prior reports, adding the costimulatory agent FAP-4-1BBL to CEACAM5 x CD3 TCB did not enhance ex vivo cytokine induction compared to the TCB alone^9,30^. This lack of observed synergy may reflect the 48h evaluation window, which is insufficient to capture qualitative T cell changes, and the inability of EVOC to capture dynamic immune cell recruitment. Finally, while FAP is characteristic of CRC, NSCLC and PDAC stroma, its expression was not directly quantified in these specific tissue fragments.

Several limitations of this work must be acknowledged, including the modest sample size of the clinical cohort and the EVOC analysis, alongside the lack of additional data due to early trial cessation. Development of ex vivo assays capable of measuring direct T cell-mediated cytotoxicity and longer-term culture capabilities to interrogate distal changes in T cell phenotypes and functional states will be particularly relevant, given evidence that CD8 T cell infiltration alone does not fully predict outcome to immune-oncology therapies^26,31^. In summary, by integrating humanized mouse studies, ex vivo PD profiling, and Phase 1 clinical biomarker benchmarks, this study provides a unique, translational framework that identifies tumor immune contexture and circulating target antigen as key determinants of early TCB-induced PD response in solid tumors. These findings emphasize that antigen density alone is insufficient to predict functional reactivity and underscore the value of human-centric models that better reflect inter-patient heterogeneity and PD effect size to develop translational biomarker strategies.

## Methods

### Study design and rationale

An integrated translational framework combining humanized mouse studies, patient-derived ex vivo tumor explants, and clinical biomarker analyses was used to characterize early pharmacodynamic activity of CEACAM5-directed CD3 T cell bispecific antibodies (CEACAM5 x CD3 TCB) (Fig. 1B).

### Humanized mouse studies

The humanized animal model has been previously described^32^. All animal experiments were conducted in accordance with institutional and governmental regulations and were approved by the Veterinary Office of the Canton of Zurich (license ZH193/2014), the institutional ethics and licensing authority overseeing this work. Mice were housed under specific-pathogen-free conditions with controlled temperature, humidity, and a 12-h light/dark cycle, and had ad libitum access to food and water. Mice were treated with drugs or controls intravenously in conscious, gently restrained manner without anesthesia, consistent with standard practice. Prior to injection, tails were prewarmed with a heat lamp to induce vasodilation and disinfected with 70% ethanol. Mice were humanely euthanized at the study endpoint, or earlier if signs of sickness or suffering were observed, prior to tumor harvest for flow cytometric and cytokine/chemokine analysis. Euthanasia was performed via isoflurane anesthesia followed by cervical dislocation and confirmatory exsanguination, in accordance with AVMA guidelines and institutional standard operating procedures. Mice were implanted subcutaneously with MKN45 human gastric carcinoma cells and randomized to treatment groups once tumors reached approximately 150–200 mm³. CEACAM5 x CD3 TCB or vehicle control (sterile histidine buffer) was administered intravenously once weekly for six consecutive weeks. Histidine buffer was selected as the control to allow for cross-comparability between multiple therapeutic modalities while adhering to animal conservation guidelines. Tumor growth was monitored by caliper measurements, and animals were assessed daily for clinical signs and adverse events.

For intratumoral PD assessment, a subset of mice (*n* = 4 per group) was sacrificed at 24 h post-second dose for flow cytometric analysis and for cytokine and chemokine analysis. The cohort size was selected based on the expected high consistency of PD data in the humanized mouse model. Results were compared against vehicle-treated controls to determine treatment-induced changes.

### Tumor dissociation and single-cell preparation

Subcutaneous tumors of four mice per treatment condition were harvested from humanized mice and weighed immediately following extraction before immersion in sterile ice-cold phosphate-buffered saline (PBS). Tissue dissociation was performed using the gentleMACS™ system (Miltenyi Biotec) according to a modified manufacturer’s protocol. Briefly, tumors were transferred into gentleMACS C-tubes containing 5 ml RPMI-1640 medium supplemented with Collagenase D (1 mg/ml; Roche Diagnostics) and DNAse I (0.025 mg/ml; Sigma-Aldrich). Samples were processed using the gentleMACS program m_imptumor_01_01, followed by a 30-min incubation at 37 °C with mechanical mixing every 10 min. A second dissociation step was performed using the program m_imptumor_02_01. The resulting cell suspension was filtered through a 70 µm nylon cell strainer (BD Falcon), washed twice, and resuspended in FACS buffer (DPBS without Ca^2+^/Mg^2+^, supplemented with 2% FCS and 2 mM EDTA).

### Flow cytometry

Single-cell suspensions were transferred to 96-well plates (approximately 100 µl per sample) and centrifuged at 1450 rpm for 5 min at 4 °C. The supernatant was discarded, and cells were resuspended in 100 µl of an antibody cocktail prepared in FACS buffer. To prevent non-specific binding, the master mix contained a purified rat anti-mouse CD16/CD32 Fc-block (clone 2.4G2; BD Biosciences, 553142) alongside the following human-specific fluorophore-conjugated antibodies: AlexaFluor 488-anti-CD45 (clone HI30), BV605-anti-CD3 (clone OKT3), APC-Cy7-anti-CD4 (clone OKT4), and AlexaFluor 700-anti-CD8 (clone SK1); all antibodies were sourced from BioLegend/Lucerna-Chem.

Samples were incubated in the dark for 30 min at 4 °C. Following incubation, cells were washed with 200 µl FACS buffer and stained with DAPI (4’,6-diamidino-2-phenylindole; Roche, Cat. no. 10236276001) for dead cell exclusion according to the manufacturer’s instructions. Data were acquired using a BD LSRFortessa™ cell analyzer (BD Biosciences). Manual gating and analysis were performed using FlowJo software v.10.4 (FlowJo, LLC). Tumor-infiltrating immune cells were identified as living (DAPI^-^) CD45^+^ cells, with further gating to define CD8^+^ and CD4^+^ T cell subpopulations. For the quantification of intratumoral T cells, following staining and flow cytometric acquisition, raw flow cytometry counts were adjusted by the dilution factors applied. These counts were normalized to the initial tissue weight to express infiltration density as cells per milligram (mg) of tumor tissue to account for variations in tumor size at the time of harvest, enabling standardized comparison across samples.

### Cytokine and chemokine analysis

Small tumor fragments were snap-frozen in liquid nitrogen and stored at −80 °C. Whole protein was isolated by homogenizing tissues in Bio-Plex® Cell Lysis Buffer supplemented with EASYpack Protease Inhibitor Cocktail (Roche) using a Precellys®24 Homogenizer. Protein concentrations were determined using the BCA™ Protein Assay Kit (Thermo Scientific). Quantitative analysis of 40 human cytokines and chemokines was performed using the Bio-Plex Pro™ Human Chemokine Panel (Bio-Rad Laboratories) on a Bio-Plex system. Final cytokine concentrations were normalized to the total protein content and are expressed as pg/mg of protein.

### Patient samples and ex vivo tumor explant culture

Fresh tumor tissue was obtained from surgical resections of consenting patients with CRC, PDAC or NSCLC (*N* = 14; Table 1). Tumor specimens were transported in preservation medium and processed within 2 to 6 h of resection.

Tumor samples were sectioned into approximately 300 µm thick slices using a vibratome and cultured as organotypic tumor explants under conditions that preserve tissue architecture and immune cell viability, as previously described. Two to three explant slices randomly assigned per condition were cultured for up to 72 h.

Explants were treated with a CEACAM5 x CD3 TCB either alone or in combination with a FAP 4-1BBL costimulatory agent. Control conditions included an untargeted TCB (DP47 × CD3 TCB) or medium alone. Culture supernatants were collected at 48 h and stored at −80 °C for cytokine analysis. Corresponding tissue slices were fixed in formalin for histological and immunohistochemical evaluation.

### Biomarker analysis of ex vivo explants

Formalin-fixed paraffin-embedded (FFPE) tumor explants were serially sectioned and stained with hematoxylin and eosin (H&E) to assess tissue morphology. Immunohistochemistry (IHC) was performed using antibodies directed against CD3 to quantify T cell infiltration and CEA to assess antigen expression. Whole-slide digital scanning and quantitative image analysis were used to determine CD3 T cell quantities using QuPath software. In short, tissue sections were analyzed using the positive cell detection function. Cells were first identified based on nuclear detection parameters, after which CD3 staining intensity was assessed, and cells were classified as positive or negative according to predefined thresholds. The percentage of CD3-positive cells was calculated as the number of CD3-positive cells divided by the total number of detected cells within the analyzed region of interest. CEA expression was evaluated using a semiquantitative H-score. All analyses were performed using identical detection and classification parameters across samples to ensure consistency and comparability. Cytokine concentrations in explant culture supernatants were quantified using the ProcartaPlex assays (Thermo Fisher Scientific) and read on a Luminex instrument according to manufacturer instructions. Analytes included interferon gamma, interleukin-2 receptor alpha, tumor necrosis factor alpha, interleukin-10, interleukin-6, interleukin-8, and granzyme B. Due to limitations in tissue and sample availability, not all 14 tumor samples were subject to the full set of IHC and cytokine-based biomarker analysis.

### Clinical trial patient samples

Clinical biomarker analyses were performed using samples obtained from patients enrolled in a first-in-human, open-label, multicenter, dose-escalation Phase 1 clinical study of CEACAM5 x CD3 TCB (ClinicalTrials.gov identifier NCT03539484, registration date 29/05/2018). The study was approved by Videnskabsetiske Komitéer for Region Hovedstade, Hillerød, Denmark; CEIC Hospital Vall D’Hebron, Barcelona, Spain; and Independent Belgian Ethics Committee, Leuven, Belgium, as well as local ethics and governance committees. The study was conducted in accordance with the Declaration of Helsinki, the International Council for Harmonization Good Clinical Practice guidelines, and all applicable local and national regulations. Institutional review boards at each study site approved the study, and all patients provided written informed consent.

Patients received intravenous infusions of the drug every three weeks, at dose levels ranging from 400 µg to 1800 µg (Table 2). Peripheral blood samples included in this study were collected prior to treatment and longitudinally for pharmacokinetic and pharmacodynamic biomarker analyses at 2, 24, 48 and 168 h following the first dose. Fresh paired tumor biopsies were obtained prior to treatment and on treatment at Cycle 2 Day 8 (pre-dose) for characterization of the TME and were formalin fixed and paraffin embedded.

### Clinical biomarker analyses

FFPE tissue blocks were obtained and sectioned consecutively for H&E and IHC, which were performed at Discovery Life Sciences (Kassel, Germany) and CellCarta (Antwerp, Belgium). Samples passing quality control for sufficient tumor content without necrotic areas were included in the analysis. Tumor biopsies were assessed by immunohistochemistry for CD8-positive T cells and CEA expression. CD8⁺ T-cells were scored visually using a validated multiplex PanCK-CD8 assay (Clone SP239 and Clone AE1/AE3/PCK26). This dual staining approach facilitated the classification of tumors based on the spatial location and density of CD8⁺ T cells within tumor intraepithelial (IE) and intratumoral stroma (ITS) compartments. Using this approach, samples were categorized into three phenotypes: Inflamed (IE2 + IE3 ≥ 20%), Excluded (ITS2 + ITS3 > 20% and IE0 + IE1 ≥ 80%), or Desert (IE0 + IE1 ≥ 80% and ITS0 + ITS1 ≥ 80%)^20^ (Fig. S4). Scoring was performed by CellCarta pathologists. This approach has been extensively validated for use in biopsy samples. CEA expression was evaluated using Cell Marque CEA31 monoclonal antibody. Staining was evaluated by a pathologist to determine the percentage of positive tumor cells at varying intensities (1+, 2+, 3+), which were integrated into a weighted H-score (range 0–300) calculated as (% cells at 1+) × 1 + (% cells at 2+) × 2 + (% cells at 3+) × 3. Tumor tissue sections with at least 1+ tumor cells with a level of 1 + CEA staining were considered CEA positive.

Bulk RNA sequencing was performed on paired baseline and on-treatment tumor biopsies to assess immune-related transcriptional programs. FFPE tissue was macrodissected using H&E as a guide to enrich tumor content. RNA was extracted using the High Pure FFPE RNA isolation kit (Roche), assessed by Qubit and Agilent Bioanalyzer for quantity and quality. The prepared libraries were sequenced using the Illumina platforms. RNA-seq data were analyzed as previously described^24^. Downstream analyses focused on pathway-level immune signatures (refer to Table S1 for gene signature list). Signature scores were estimated via rank-biserial values (BioQC V.3.15) and compared using the Wilcoxon test.

Serum and plasma samples were collected and processed centrally for measurement of soluble CEA and circulating cytokines using ELISA and multiplex ProteinSimple immunoassays on an Ella platform (R&D Systems).

### Statistical analysis

All statistical analyses were performed using R software (version 4.2.0). For the humanized mouse study, differences in tumor volume, cytokine concentrations and immune cell densities between treatment groups were evaluated using the Wilcoxon rank-sum test. For the ex vivo explant study, cytokine concentrations were compared between treatment groups using the Wilcoxon signed-rank test; additionally, cytokine responses were assessed by calculating log-transformed treatment-to-control ratios and testing their deviation from zero using the Wilcoxon signed-rank test within CD3 low and CD3 high subgroups. For the phase I study, cytokine concentrations were compared across timepoints, and fold changes at 24h were compared between desert and excluded/inflamed tumors, both using the Wilcoxon signed-rank test. Variability in cytokine response was quantified using the CV, calculated on the original concentration scale. Associations between baseline intratumoral T cell density and cytokine induction were evaluated using Spearman’s rank correlation. All statistical tests were two-sided and a *p*-value of less than 0.05 was considered statistically significant.

## Supplementary information

Supplementary information

## Data Availability

Qualified researchers may request access to individual participant–level data through the clinical study data request platform (https://vivli.org/ourmember/roche/). Further details on F. Hoffmann-La Roche Ltd’s criteria for eligible studies are available at https://vivli.org/members/ourmembers/. For further details on F. Hoffmann-La Roche Ltd’s Global Policy on the Sharing of Clinical Information and the procedure to request access to related clinical study documents, see https://www.roche.com/innovation/process/clinical-trials/data-sharing.
