# Supplementary material for: Early functional reactivity to a CEACAM5 x CD3 T cell bispecific antibody in solid tumors associates with baseline immune contexture rather than target antigen density alone
Source: NPJ Precis Oncol. 2026 Aug 14;10:394. doi: 10.1038/s41698-026-01643-3 (PMC13672796; doi:10.1038/s41698-026-01643-3)
Supplement: Supplementary file 1 [file 41698_2026_1643_MOESM1_ESM.pdf]

Table S1. Inter-subject variability of cytokine responses across preclinical and clinical settings

Cytokine	CV (%)		
	Mouse	EVOC	Phase I Patients
IL-2	55	86	—
IL-6	64	115	158
IL-8	44	78	121
IL-10	36	120	157
IFNγ	52	135	190
TNFα	30	103	79

Coefficient of variation (CV) values for cytokine induction following CEACAM5×CD3 TCB treatment are shown for humanized mouse models, patient-derived *ex vivo* organ cultures (EVOC), and patients treated in a Phase I clinical study. Clinical variability was assessed using cytokine levels induced during the first cycle of treatment (Cycle 1). CV values are reported as percentages, with higher values indicating greater inter-patient heterogeneity. Dashes indicate cytokines not available for analysis in the clinical dataset.

Table S2. Immune-related signatures and their constituent genes

Gene signature	Gene list
IFNg pathway	*IFNG, CXCL9, CXCL10, STAT1*
Effector T cell	*CD8A, GZMA, GZMB, IFNG, EOMES, PRF1, CXCL9, CXCL10, TBX21*
Regulatory T cell[1]	*FOXP3, CCR8, TNFRSF18, LAYN, IKZF2, RTKN2, CTLA4, BATF, IL21R*
IL2 response[2]	*S100A9, IGSF9, FCGR3B, F5, SDC1, CYP1B1, ATP6V1B1, IL1RN, STEAP3, TUBA3D, COL4A4, PCOLCE2, CP, CXCL2, EREG, GZMA, TREM1, ZNF853, MUC17, FCN1, LRRN4CL, NNMT, B4GALNT3, GJB2, AQP9, MEFV, SHISA9, HP, CCL2, HLF, CD300E, TJP3, ANGPTL4, FPR2, LILRA5, APOL1, APOBEC3A, MAP7D2, SLITRK4*
Antigen processing	*TAPBP, TAP1, TAP2, PSMB9, PSMB8*
HALLMARK_TGF_BETA_SIGNALING	54 genes, GSEA link
HALLMARK_G2M_CHECKPOINT	200 genes, GSEA link
HALLMARK_E2F_TARGETS	200 genes, GSEA link
HALLMARK_EPITHELIAL_MESENCHYMAL_TRANSITION	200 genes, GSEA link
HALLMARK_WNT_BETA_CATENIN_SIGNALING	42 genes, GSEA link
HALLMARK_HEDGEHOG_SIGNALING	36 genes, GSEA link
HALLMARK_ANGIOGENESIS	36 genes, GSEA link
HALLMARK_HYPOXIA	200 genes, GSEA link
HALLMARK_GLYCOLYSIS	200 genes, GSEA link
HALLMARK_COAGULATION	138 genes, GSEA link

1. Zhang, L., *et al.* Lineage tracking reveals dynamic relationships of T cells in colorectal cancer. *Nature* **564**, 268-272 (2018).
2. Eisner, J.R., *et al.* Distinct Predictive Immunogenomic Profiles of Response to Immune Checkpoint Inhibitors and IL2: A Real-world Evidence Study of Patients with Advanced Renal Cancer. *Cancer Res Commun* **2**, 894-903 (2022).

Figure S1

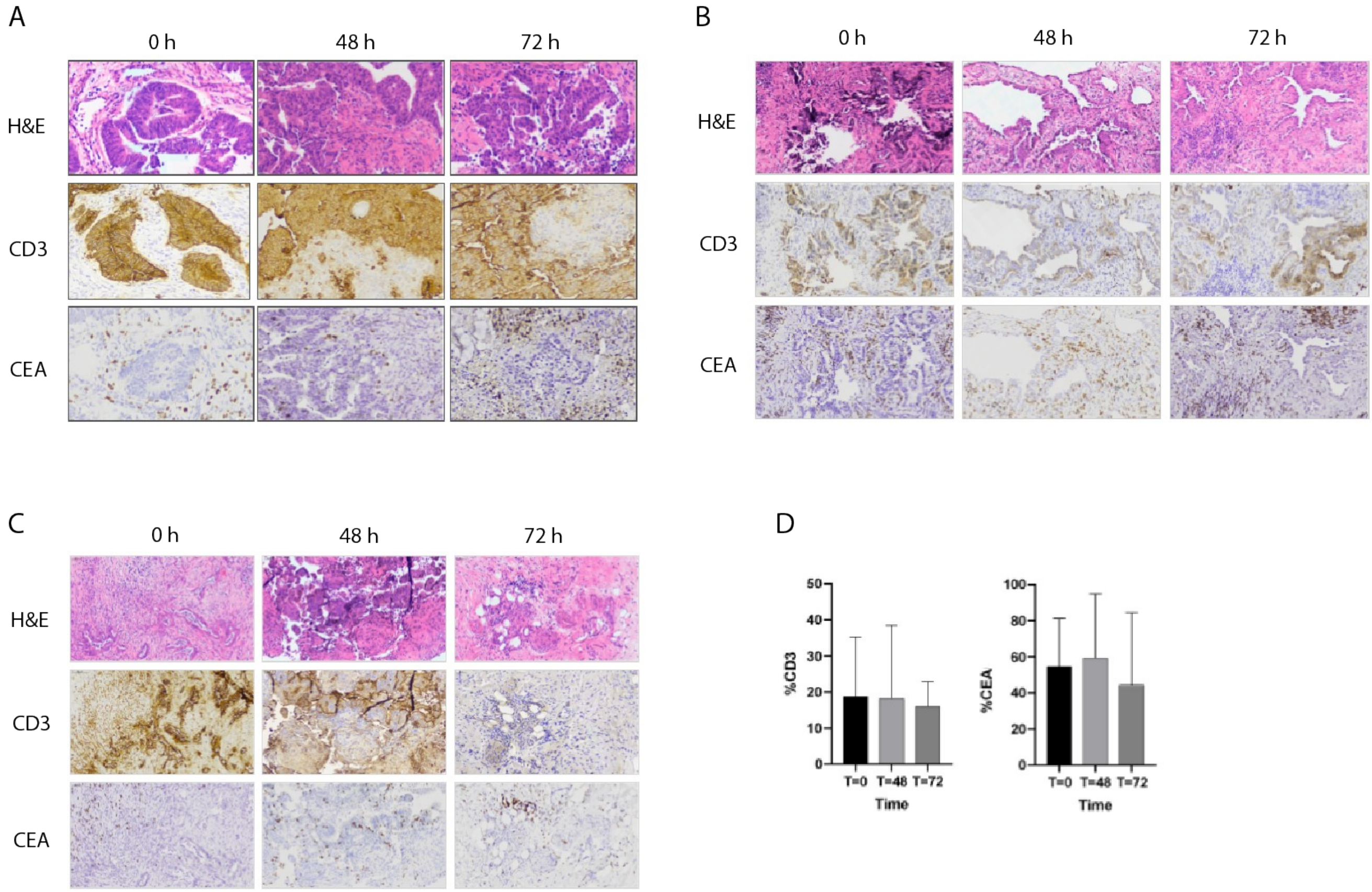
A
0 h
48 h
72 h
H&E
CD3
CEA
B
0 h
48 h
72 h
H&E
CD3
CEA
C
0 h
48 h
72 h
H&E
CD3
CEA
D
%CD3
50
40
30
20
10
0
T=0
T=48
T=72
Time
%CEA
100
80
60
40
20
0
T=0
T=48
T=72
Time

Figure S2

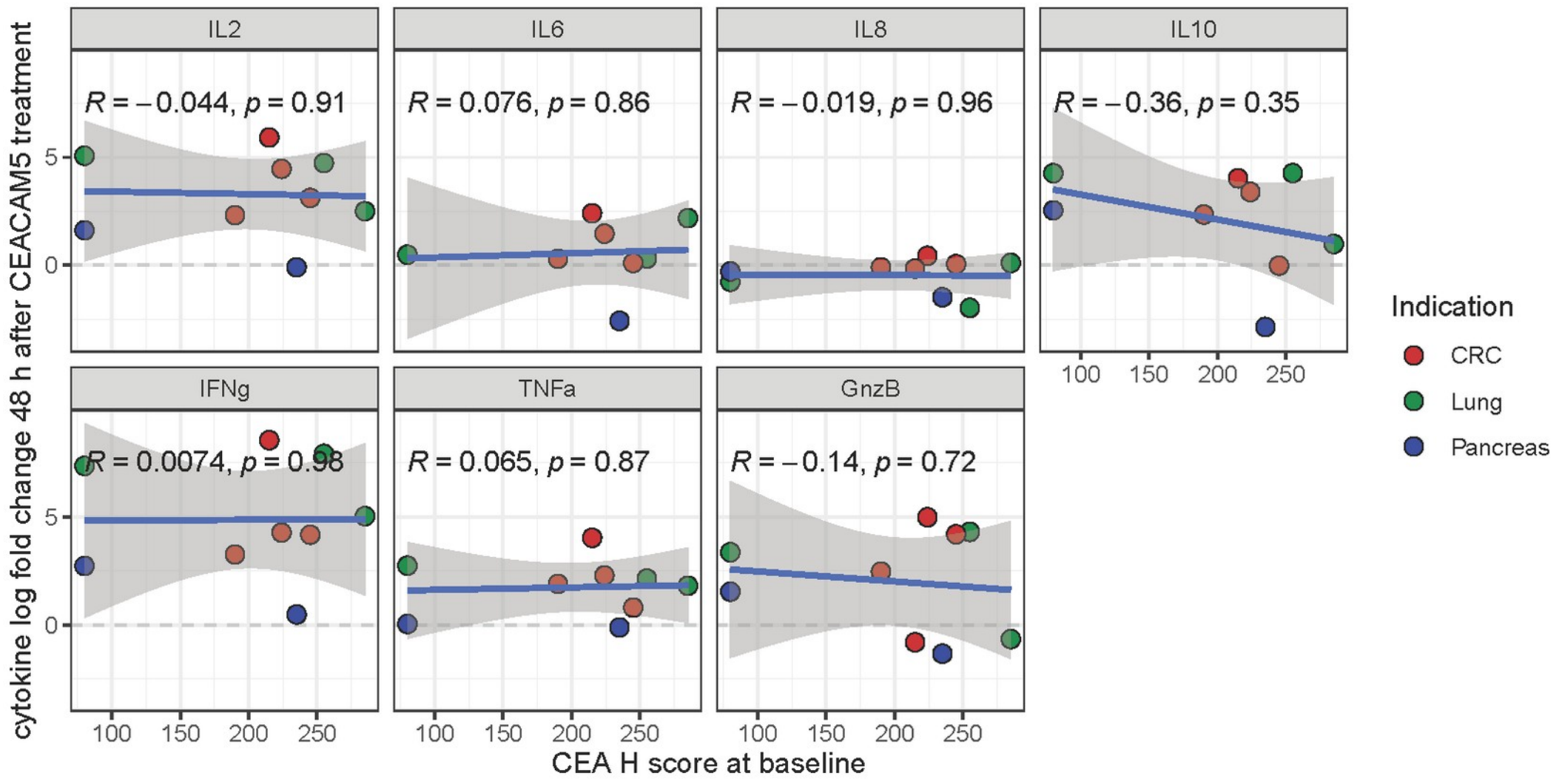

IL2
IL6
IL8
IL10
R = −0.044, p = 0.91
R = 0.076, p = 0.86
R = −0.019, p = 0.96
R = −0.36, p = 0.35
IFNg
TNFa
GnzB
R = 0.0074, p = 0.98
R = 0.065, p = 0.87
R = −0.14, p = 0.72
cytokine log fold change 48 h after CEACAM5 treatment
CEA H score at baseline
Indication
CRC
Lung
Pancreas

Figure S3

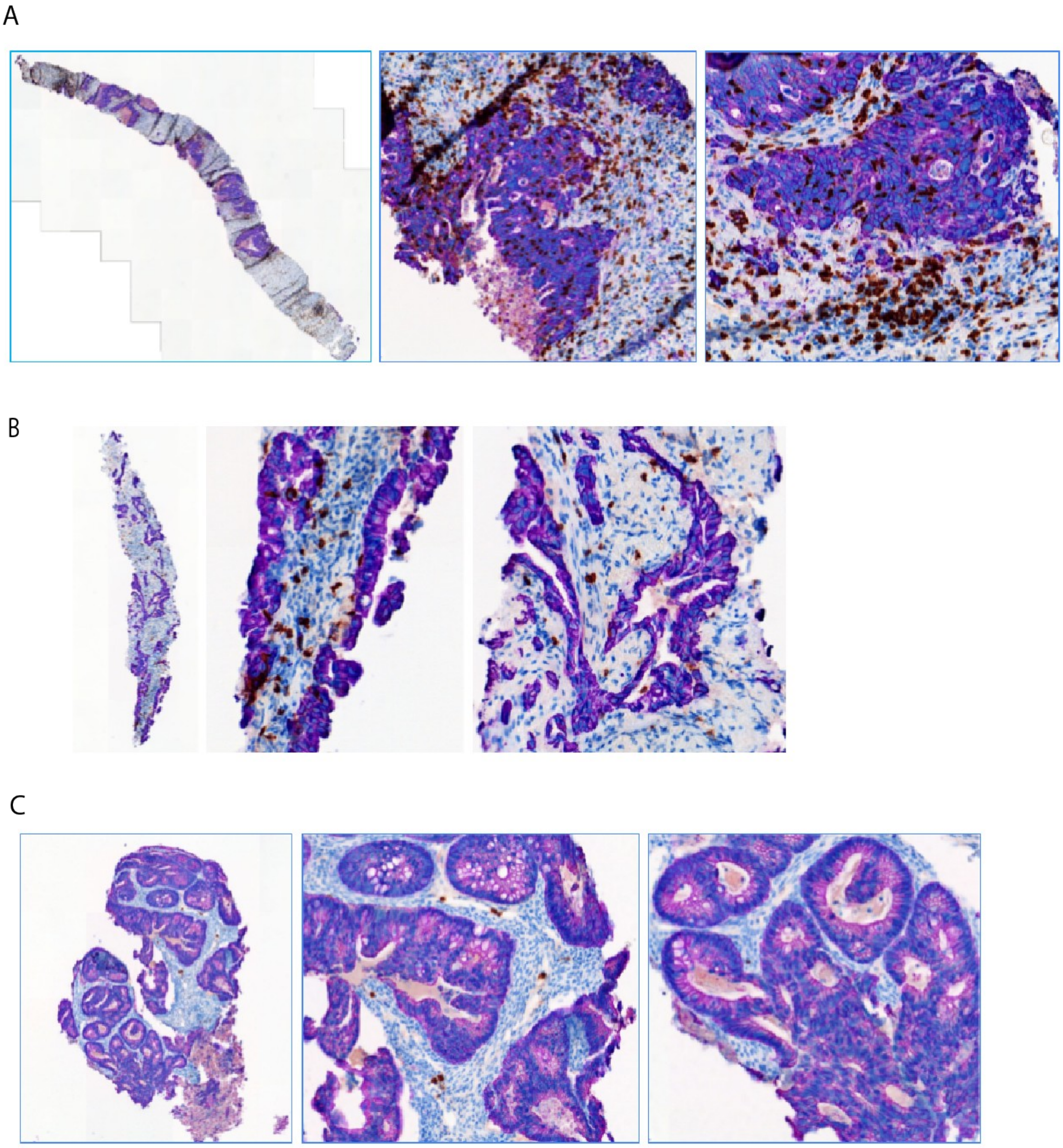

Figure S4

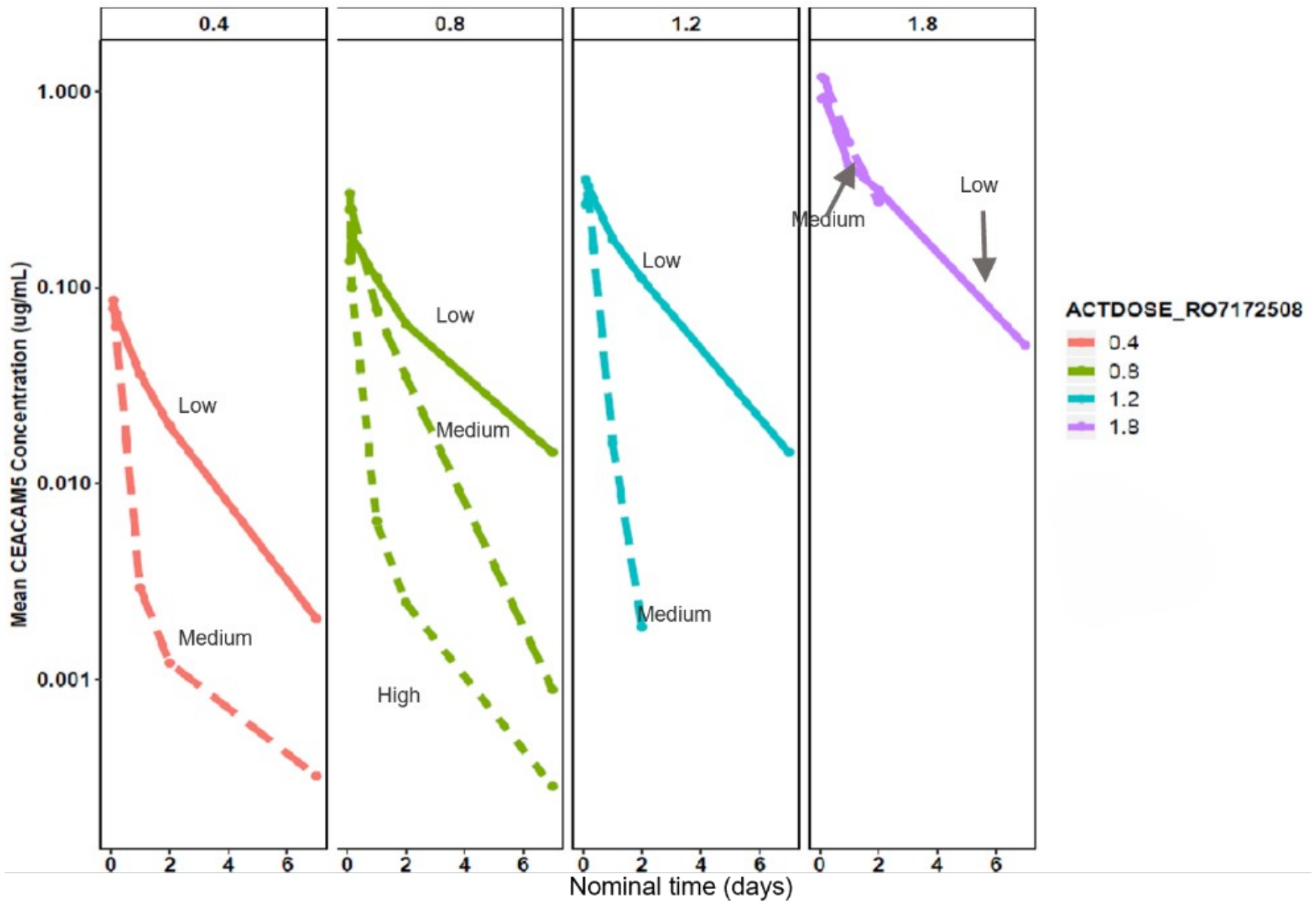

0.4
0.8
1.2
1.8
1.000
0.100
0.010
0.001
Mean CEACAM5 Concentration (ug/mL)
Low
Medium
High
0
2
4
6
Nominal time (days)
ACTDOSE_RO7172508

Figure S5

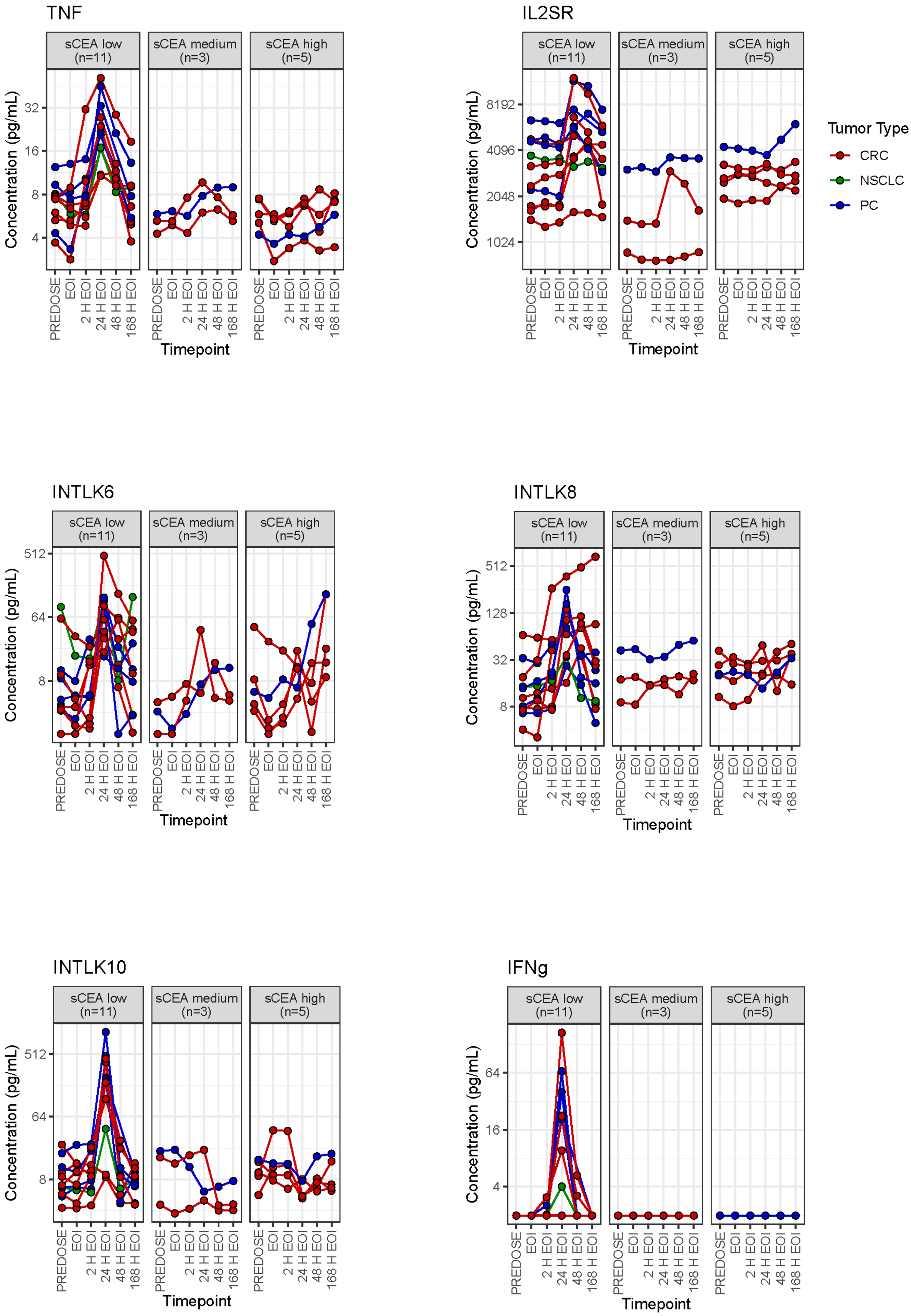
TNF
IL2SR
INTLK6
INTLK8
INTLK10
IFNg
sCEA low (n=11)
sCEA medium (n=3)
sCEA high (n=5)
Concentration (pg/mL)
Timepoint
PREDOSE
EOI
2 H EOI
24 H EOI
48 H EOI
168 H EOI
4
8
16
32
64
128
512
1024
2048
4096
8192
Tumor Type
CRC
NSCLC
PC

Figure S6

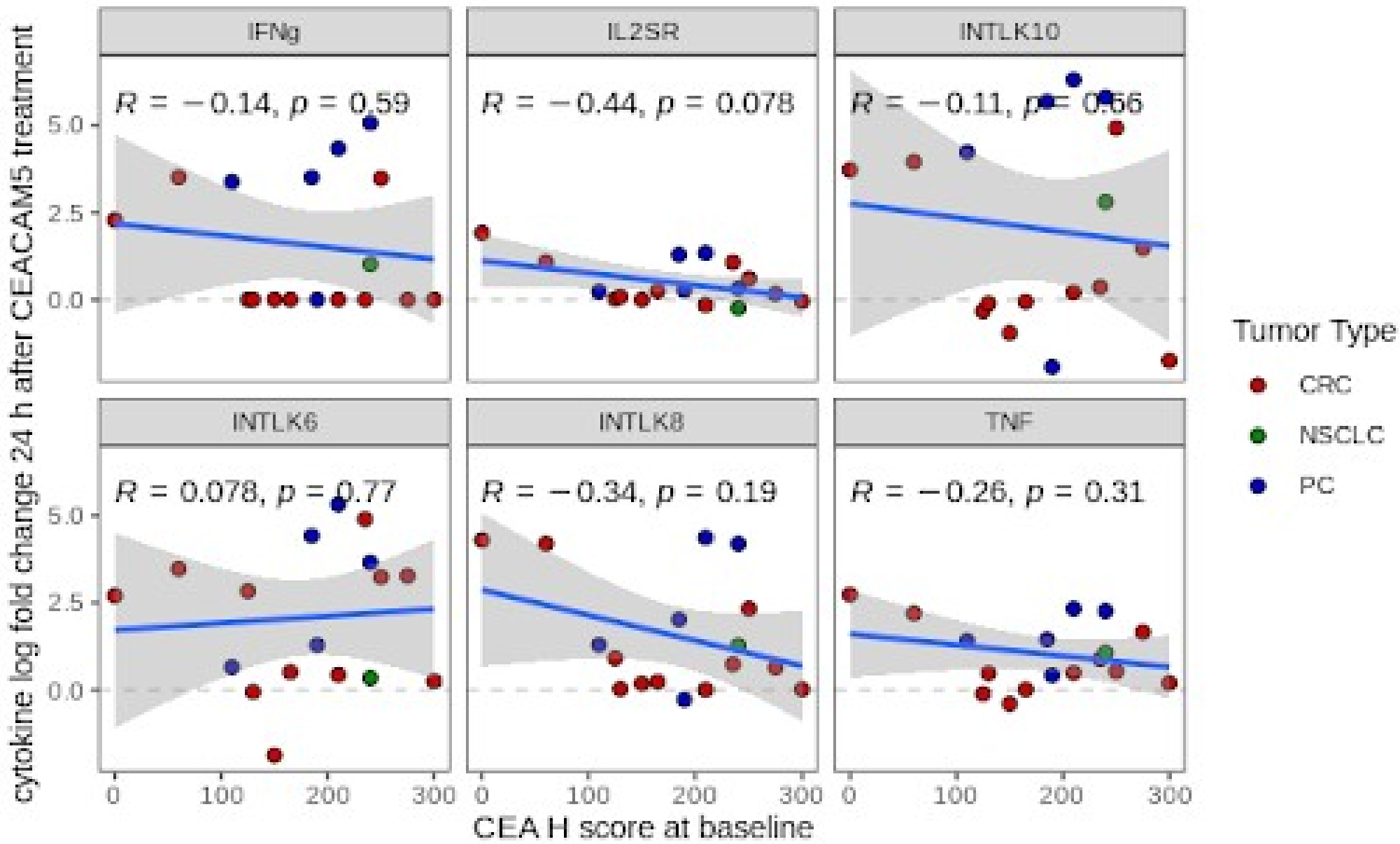
IFNg
IL2SR
INTLK10
INTLK6
INTLK8
TNF
R = −0.14, p = 0.59
R = −0.44, p = 0.078
R = −0.11, p = 0.66
R = 0.078, p = 0.77
R = −0.34, p = 0.19
R = −0.26, p = 0.31
cytokine log fold change 24 h after CEACAM5 treatment
CEA H score at baseline
Tumor Type
CRC
NSCLC
PC

Figure S7

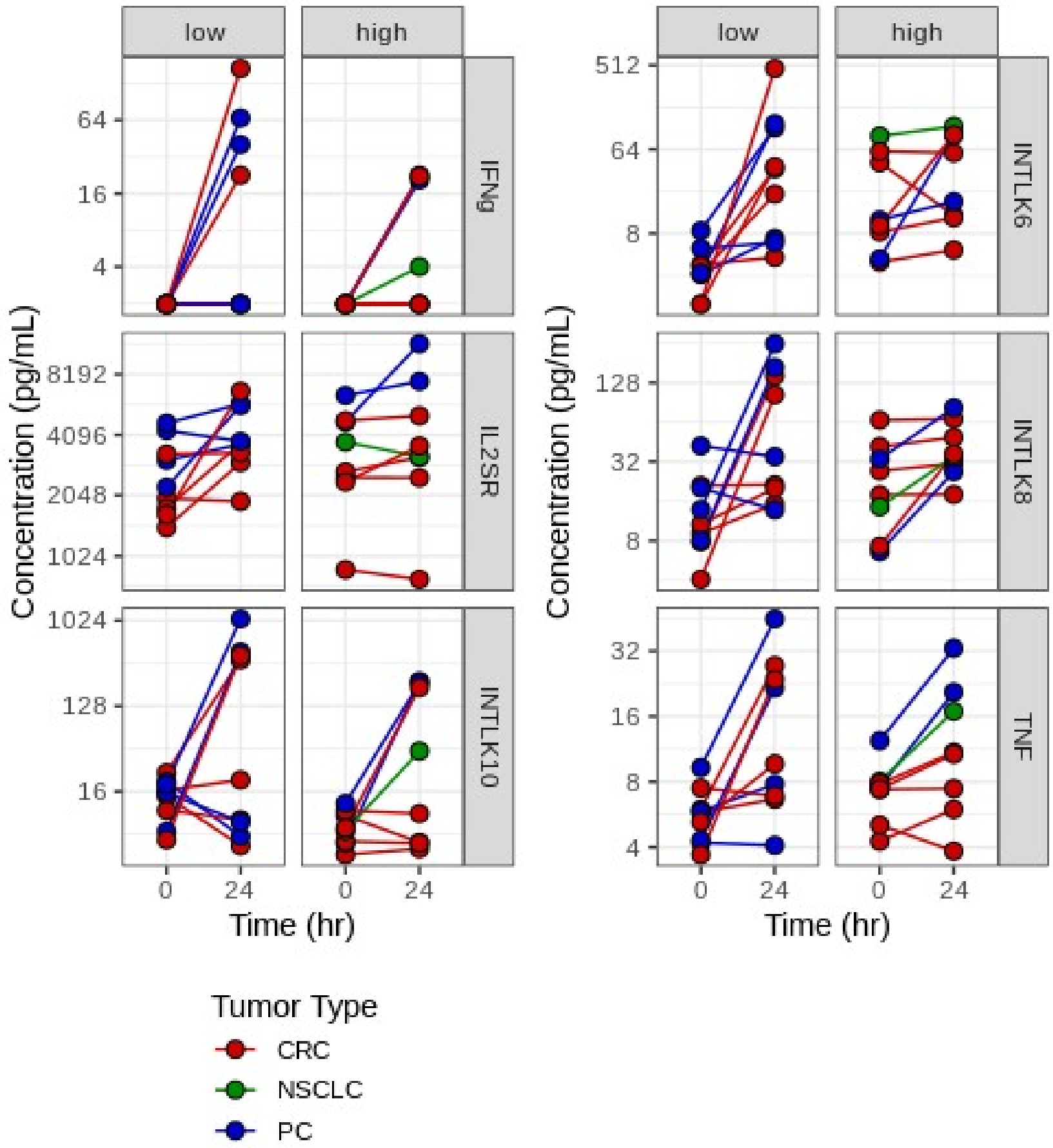

low
high
IFNg
IL2SR
INTLK10
INTLK6
INTLK8
TNF
Concentration (pg/mL)
Time (hr)
Tumor Type
CRC
NSCLC
PC

Figure S8

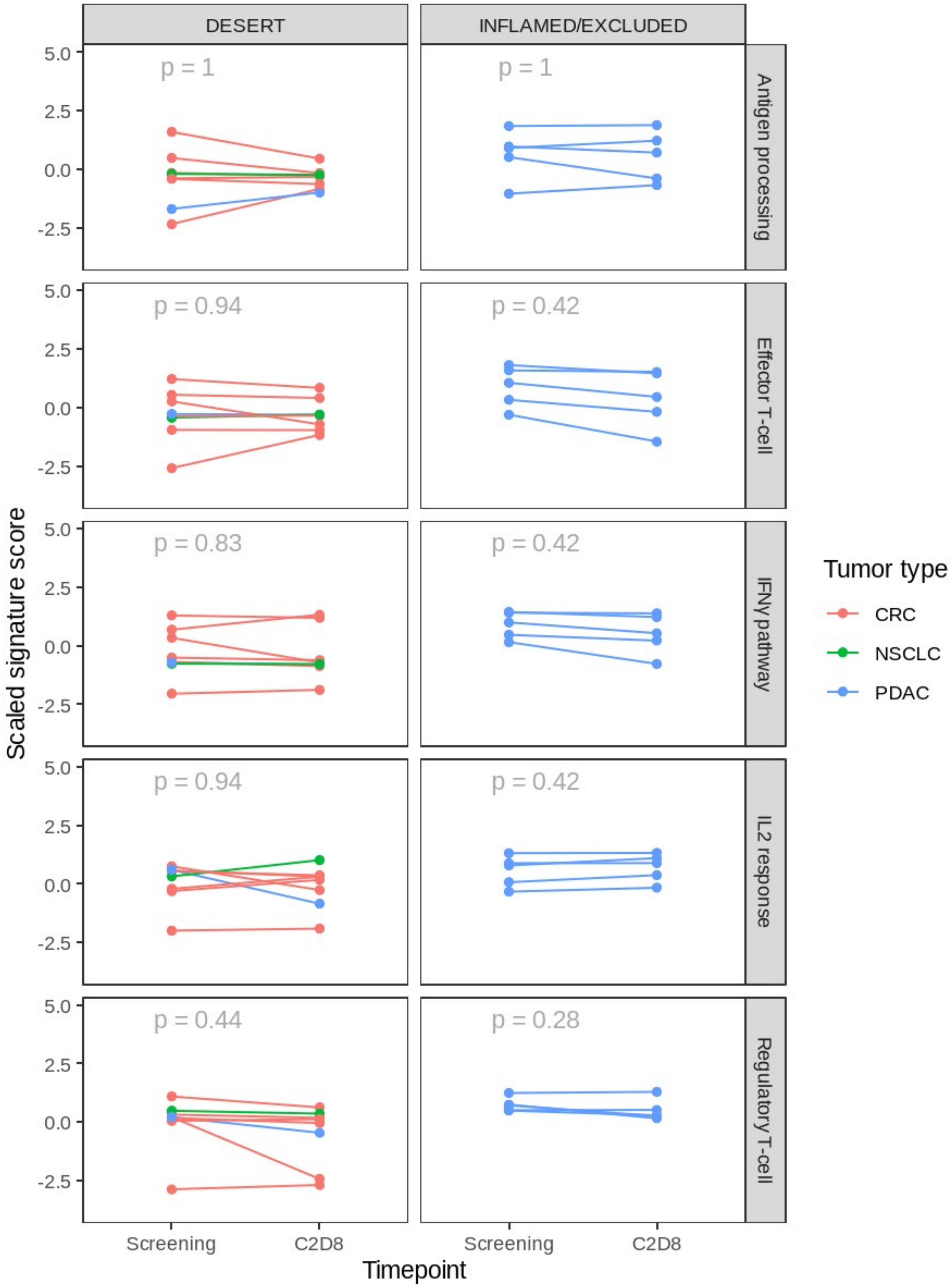
DESERT
INFLAMED/EXCLUDED
Antigen processing
Effector T-cell
IFNγ pathway
IL2 response
Regulatory T-cell
p = 1
p = 1
p = 0.94
p = 0.42
p = 0.83
p = 0.42
p = 0.94
p = 0.42
p = 0.44
p = 0.28
Scaled signature score
5.0
2.5
0.0
-2.5
Screening
C2D8
Timepoint
Tumor type
CRC
NSCLC
PDAC

Figure S9

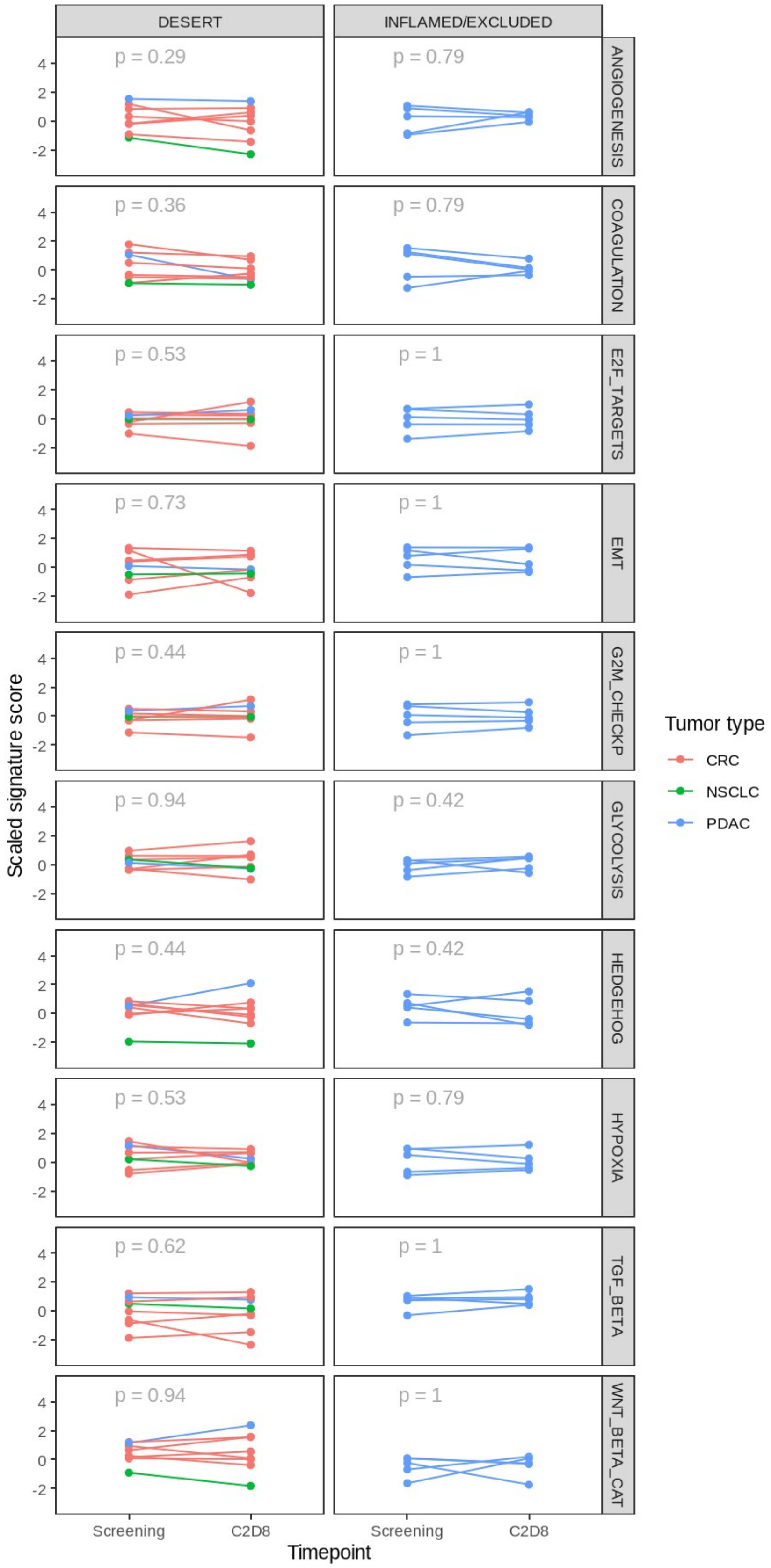

DESERT
INFLAMED/EXCLUDED
ANGIOGENESIS
COAGULATION
E2F_TARGETS
EMT
G2M_CHECKP
GLYCOLYSIS
HEDGEHOG
HYPOXIA
TGF_BETA
WNT_BETA_CAT
p = 0.29
p = 0.79
p = 0.36
p = 0.79
p = 0.53
p = 1
p = 0.73
p = 1
p = 0.44
p = 1
p = 0.94
p = 0.42
p = 0.44
p = 0.42
p = 0.53
p = 0.79
p = 0.62
p = 1
p = 0.94
p = 1
Scaled signature score
Screening
C2D8
Timepoint
Tumor type
CRC
NSCLC
PDAC

Figure S10

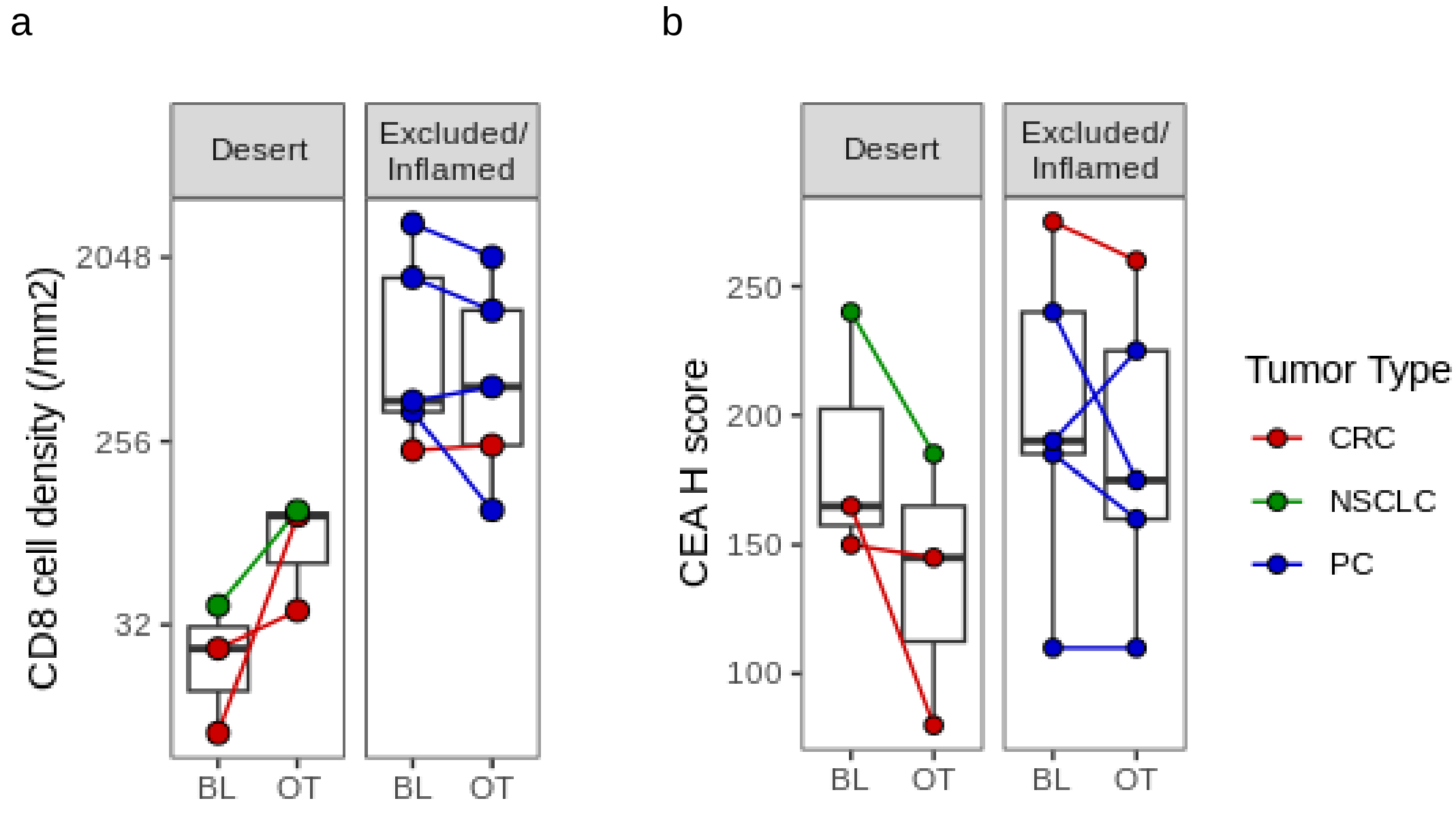
a
b
Desert
Excluded/
Inflamed
CD8 cell density (/mm2)
2048
256
32
BL
OT
CEA H score
250
200
150
100
Tumor Type
CRC
NSCLC
PC

Supplementary Figure Legends

Figure S1. Preservation of tissue integrity and heterogeneity in ex vivo organ culture (EVOC). Baseline and *ex vivo* cultured tissue sections at 0h, 48h and 72h from (a) colorectal carcinoma (b) non-small cell lung cancer and (c) pancreatic adenocarcinoma patient tumors. H&E sections (top row) demonstrate preserved morphology over time; intratumoral stroma retains a viable microenvironment, including fibroblasts, vessels, and infiltrating immune cells, across all timepoints. CEA staining (middle row) primarily reveals membranous expression, with varying intensity and less cytoplasmic staining. The absence of stromal or background CEA staining confirms a tumor-specific pattern. CD3 immunostaining (bottom row) shows specific expression restricted to lymphocytes, with no background staining. The overall morphology of the tumor and stroma is preserved, supporting marker stability during ex vivo culture. Samples represented at each timepoint originate from different regions of the original tumor.

Figure S2. Baseline CEA expression does not associate with cytokine induction in *ex vivo* organ cultures (EVOC). Scatter plots showing the relationship between baseline tumor CEA expression, quantified by H-score, and induction of IFNγ, IL-2, IL-10, TNFα, IL-6, Granzyme B and IL-8 at 48 hours following CEACAM5×CD3 TCB treatment of patient tumors *ex vivo*. Each point represents an individual patient tumor sample, colors represent indication.

Figure S3. Example of CD8 immune phenotype classification of Phase 1 patient biopsy samples. Images of CD8/panCK-stained immunohistochemistry analysis of tumor tissues obtained from patients with distinct immune phenotypes. (a) CD8 inflamed phenotype with a diffuse infiltration of high-density patches penetrating the intra-epithelial compartment (IE0+IE1 <80% and IE2+IE3 $\geq$20%) and the intra-stromal compartment (ITS0 + ITS1 < 80% and ITS2 + ITS3 > 20%) defines and inflamed phenotype. This profile indicates a high density of CD8+ T cells actively infiltrating the tumoral epithelium, paired with a high density of immune cells in the surrounding intratumoral stroma. (b) CD8 excluded phenotype with an accumulation of low-density patches in the intra-epithelial compartment (IE0 + IE1 $\geq$ 80% and IE2 + IE3 < 20%) and high-density patches in the intra-stromal compartment (ITS0 + ITS1 < 80% and ITS2 + ITS3 > 20%) defines an immune excluded phenotype. This profile indicates low or absent CD8+ T cell infiltration within the tumor epithelium, paired with a higher density of immune cells in the surrounding intratumoral stroma. (c) CD8 desert phenotype with an accumulation of low-density patches in both compartments (IE0 + IE1 $\geq$ 80%) defines an immune desert phenotype. This profile indicates a complete absence of CD8+ T-cell infiltration within the tumor epithelium, paired with a scarcity of immune cells throughout the surrounding intratumoral stroma. IE, intra-epithelial; ITS, intra-stromal.

Figure S4. Baseline soluble CEA influences CEACAM5×CD3 TCB exposure in patients on trial. Active drug exposure across dose levels stratified by baseline soluble CEA (sCEA) concentrations (low <70 ng/mL, medium 71-100 ng/mL or high >100 ng/mL) in patients enrolled in the Phase 1 trial of CEACAM5×CD3 TCB (ClinTrials.gov identifier: NCT03539484), highlighting reduced active drug exposure in patients with elevated baseline sCEA at lower doses.

Figure S5. Peripheral cytokine kinetics following CEACAM5×CD3 TCB treatment in patients on trial. Longitudinal plasma cytokine concentrations measured at baseline and multiple post-dose timepoints relative to end of infusion (EOI) during the first cycle of treatment in patients enrolled in the Phase 1 trial of CEACAM5×CD3 TCB, panelled by baseline soluble CEA (sCEA) concentrations (low <70 ng/mL, medium 71-100 ng/mL or high >100 ng/mL). Individual patient cytokine trajectories illustrating inter-patient variability in systemic cytokine induction; color indicates indication.

Figure S6. Tumor CEA expression does not correlate with systemic cytokine responses in patients on trial. Correlation between baseline tumor CEA H-scores and fold change in IFNγ, soluble IL-2 receptor, IL-6, IL-8, TNFα, and IL-10 at 24 hours post-dose in patients enrolled in the Phase 1 trial of CEACAM5×CD3 TCB. R denotes the Spearman correlation with corresponding p-value; color indicates indication.

Figure S7. Peripheral CD3+ T cell levels do not correlate with systemic cytokine responses in patients on trial. Peripheral cytokine concentrations at baseline and 24 hours after CEACAM5xCD3 TCB administration in patients with low versus high baseline peripheral CD3+ T cell levels (n=9).

Figure S8. Lack of on-treatment modulation of immune gene signatures across tumor immune phenotypes in patients on trial. Expression of immune gene signatures in immune-desert, immune-excluded, and immune-inflamed tumors in paired biopsies obtained from patients enrolled in the Phase 1 trial of CEACAM5×CD3 TCB at screening (baseline) and on-treatment (Cycle 2 Day 8 (pre-dose)). Shown p-values are based on paired Wilcoxon test; color indicates indication.

Figure S9. Lack of on-treatment modulation of gene signatures associated with tumor remodeling across tumor immune phenotypes in patients on trial. Expression of gene signatures associated with tumor remodeling in immune-desert, immune-excluded, and immune-inflamed tumors in paired biopsies obtained from patients enrolled in the Phase 1 trial of CEACAM5×CD3 TCB at screening (baseline) and on-treatment (Cycle 2 Day 8 (pre-dose)). Shown p-values are based on paired Wilcoxon test; color indicates indication.

Figure S10. Lack of on-treatment changes in CD8 T cells or CEA expression following treatment in patients on trial. Change in intratumoral (a) CD8 T cell density and (b) CEA expression, panelled by immune phenotype following treatment in patients enrolled in the Phase 1 trial of CEACAM5×CD3 TCB. Each set of connected baseline and on-treatment samples represents an individual patients' data, color indicates indication. BL, baseline; OT, on-treatment.
